# Transformer based breast cancer classification from histopathological images using sequential residual recurrent multiscale attention network

**DOI:** 10.1038/s41598-026-51422-0

**Published:** 2026-05-05

**Authors:** V. Rajeswari, N. Kathirvel, C. Kumar, Abhinandan Routray

**Affiliations:** 1 Computer Science and Technology, Karpagam College of Engineering, Coimbatore, Tamil Nadu 641032 India; 2 Information Technology, School of Computing, Vel Tech Rangarajan Dr. Sagunthala R&D Institute of Science and Technology, Avadi, Tamil Nadu 600055 India; 3 Electronics and Communication Engineering, Rathinam Technical Campus, Coimbatore, Tamil Nadu 641021 India; 4 Manipal Institute of Technology, Manipal Academy of Higher Education, Manipal, Karnataka 576104, India

**Keywords:** Squeeze ResNet, Multiscale attention, Polarized self-attention mechanism, Chaotic Chebyshev mapping, StarFish optimization, Cancer, Computational biology and bioinformatics, Mathematics and computing

## Abstract

Breast cancer is a leading cause of mortality among women globally, highlighting the need for accurate and robust diagnostic systems. This study presents a breast cancer classification based on transformer network (BrCTransNet), a novel deep learning-based framework for breast cancer classification from histopathological images. Initially, input images are preprocessed using an Adaptive Gaussian Bilateral (AGB) filter to reduce noise while preserving essential features. Feature extraction is performed using a Sequential Residual Recurrent Multiscale Attention Network (S2RMANet), which captures rich multi-scale spatial and long-range contextual features. The extracted features are then classified using Optimized Polarized Self-Attention-based Transformer (OPATransNet), a Transformer-based model that integrates a Polarized Self-Attention Module to independently learn spatial and channel-wise dependencies. To optimize the loss function, a Chebyshev StarFish Optimization (ChStF) algorithm is used for tuning OPATransNet hyperparameter, combining Chaotic Chebyshev Mapping with the StarFish Optimization Algorithm (SFOA) to enhance performance. Experimental results using the Breast Histopathology Images dataset demonstrate that the proposed BrCTransNet achieves a classification accuracy of 98.97%, recall of 98.91%, F1-score of 98.98%, specificity of 98.36%, and MSE of 0.0103 outperforming existing models. These findings confirm the superiority of the proposed model in terms of accuracy, robustness, and convergence speed, establishing its potential as a reliable tool for breast cancer diagnosis.

## Introduction

One of the most common types of cancer that affect women worldwide is breast cancer. When breast cells proliferate out of control, it occurs. It is possible to identify the particular form of breast cancer by identifying the breast cells that have developed malignancy^1^. The breast is made up of connective tissue, ducts, and lobules. The glands called lobules are in charge of making milk. The ducts serve as conduits for the movement of milk from the lobules to the nipple^2^. The fibrous and fatty materials that make up connective tissue provide support and structure. The majority of breast cancers start in the lobules or ducts. Early detection of breast cancer is critically important because it significantly improves treatment outcomes, survival rates, and quality of life for patients. When breast cancer is diagnosed at an early stage, it is usually confined to the breast or nearby lymph nodes, making it more treatable with less aggressive therapies and fewer complications^3^. It leads to a higher chance of complete remission and a reduction in the risk of metastasis, where the cancer spreads to other parts of the body. Additionally, early-stage cancers often require less invasive surgical procedures and allow for more targeted treatment options such as hormone therapy, radiation, or chemotherapy, thereby reducing the physical and emotional toll on patients^4^. From a public health perspective, early detection also reduces the overall cost burden on healthcare systems by minimizing the need for prolonged and intensive treatments associated with late-stage diagnoses^5^. With the increasing global incidence of breast cancer, early detection through screening programs, imaging technologies and computer-aided diagnostic tools becomes an essential strategy in saving lives and optimizing clinical resources^6,7^.

Histopathological images are pivotal in breast cancer classification, offering detailed visual insights into tissue morphology that are essential for accurate diagnosis^8,9^. These images, stained with hematoxylin and eosin (H&E) allow pathologists to examine cellular structures and identify abnormalities indicative of cancer^10,11^. However, manual analysis of these images is time-consuming and subject to inter-observer variability that lead to inconsistent diagnoses^12^.​ Artificial intelligence (AI), deep learning techniques, has emerged as a transformative tool in this domain^13,14^. AI models, such as convolutional neural networks (CNNs) are trained to recognize complex patterns in histopathological images that enable rapid and consistent classification of breast cancer subtypes^15^. Several researches utilized deep mutual learning approaches to enhance diagnostic performance for demonstrating the potential of AI in improving early detection rates^16^.

Compared to conventional methods, AI-based approaches offer several advantages: they reduce diagnostic time, minimize human error, and provide standardized assessments^17^. Moreover, AI can assist in areas with limited access to expert pathologists, ensuring broader reach of quality diagnostic services^18^. As AI continues to evolve, its integration into histopathological analysis promises to enhance the accuracy and efficiency of breast cancer diagnosis, contributing to better patient outcomes^19,20^.​ In this research, a transformer-based deep learning framework for accurate disease classification from histopathology images is provided. The novelty is in combining an OPATransNet to learn intricate spatial and channel dependencies, an S2RMANet to extract rich sequential spatial dependencies and long-range contextual features, and an AGB filter to effectively reduce noise and preserve edges. Furthermore, the ChStF algorithm is used to prevent local optima and improve convergence time. Compared to traditional approaches, this framework has the advantage of significantly improving diagnostic accuracy, robustness, and learning stability while lowering misclassification and computing complexity.

The major contributions of the research are:

- **BrCTransNet design:** This research presents a novel BrCTransNet framework that addresses key gaps in breast cancer histopathology analysis by combining polarized transformer-based classification, recurrent multiscale attention-based feature extraction, and adaptive Gaussian bilateral preprocessing.
- **S2RMANet:** Squeeze ResNet, GRU-based sequential modeling, and multiscale attention are combined in a novel S2RMANet to capture long-range contextual dependencies and local textures in histopathology images.
- **OPATransNet:** The proposed OPATransNet uses decoupled polarized spatial and channel self-attention, which allows for better discriminative global–local feature representation with enhanced computing efficiency for breast cancer classification, in contrast to traditional Vision Transformers.
- **Optimizing loss using ChStF:** In order to improve exploration, avoid local optima trapping, and speed convergence during transformer loss-function optimization, this study presents a novel hybrid optimization algorithm that combines Chebyshev chaotic mapping with StarFish Optimization.

The research is organized as: Section “Related works” presents the related works with problem statement and the detailed proposed methodology in Section “Proposed methodology”. The results are presented in Section “Result and discussion” and the conclusion in Section “Conclusion”.

## Related works

A number of studies have addressed this area, and this section presents a brief overview of recent work on breast cancer classification.

Enhanced shallow convolutional neural network (ES-CNN) was designed by^21^ to assist histopathologists in the early and accurate diagnosis of breast cancer. The ES-CNN model was implemented and trained on the BreakHis dataset. The architecture was shallow that indicates that the model has fewer convolutional layers, pooling layers, and fully connected layers compared to DCNNs. The design was optimized for feature extraction relevant to breast cancer classification by balancing accuracy and computational efficiency. The shallow architecture of the ES-CNN resulted in significantly lower computational costs and faster training times compared to DCNNs. However, its shallow nature may limit the capacity to capture complex hierarchical features.

Deep Vision Transformers (ViTs) and Convolutional Neural Networks (CNNs) (Deep ViT-CNN) were designed by^22^ to improve the speed and accuracy of breast cancer histopathology image classification. The designed architecture integrates CNNs for initial feature extraction and ViTs for global context understanding. The design aims to reduce the computational complexity of ViTs by minimizing input patches. In this, ConvMixer enables the extraction of relevant patches from input images to minimize the number of input patches. Faster training and inference times were accomplished by the designed model due to reduced computational demands. Despite improvements in efficiency, the model struggles with high-resolution images at larger magnifications.

Residual Network with modified U-Net architecture (ResMTUNet) was designed by^23^ to effectively capture both local and global features for accurate diagnosis of breast cancer. The extracted features were aggregated for enhancing the representational capability of the model. Attention mechanism based attention allows the model to focus on the most relevant features. Finally, K-Nearest Neighbor was employed to construct an adjacency graph of cells at the edges, capturing the spatial structure of the tumor microenvironment. The attention-based aggregator enhances the model’s ability to represent complex breast cancer tissue types. While the model outperforms other models, the achieved F1 score and accuracy remain moderate, indicating room for improvement in complex tissue representation.

Dual-Branch Adaptive Frequency Domain Fusion Network (AFFNet) was designed by^24^ for breast cancer classification. The model was designed to simultaneously capture fine-grained local details and global structural information. Then, multi-spectral channel attention was employed to effectively extract local high-saliency features from pathological images. The filtering was employed to enhance high-frequency information while preserving low-frequency features and minimizing information loss. The dual-branch design demands high computational resources, limiting its practicality in resource-constrained scenarios.

Statistical Attentions and Wavelet Aided Lightweight Network (SAWL-Net) was designed by^25^ for breast cancer classification. The designed model utilized MobileNetV2 backbone as the foundation of the SAWL-Net architecture for providing a lightweight and efficient base. The designed model considered hybrid attention mechanism to enhance feature representation. Its lightweight design makes it suitable for environments with limited computational capacity, though complex attention modules can lead to overfitting.

The Hybrid Fusion Transformer Network (HFT-Net) was suggested by^26^ for extrapolating intricate visual patterns to classify breast cancer. This approach uses a multi-head attention mechanism (MHA) to learn meaningful feature associations and enhance information interaction, producing more discriminative representations than traditional feature fusion techniques. Deep models require a lot of data, however transfer learning and fine-tuning techniques allowed for great success with less samples, and pre-trained models were adapted to facilitate an effective learning process. HFT-Net strives for balanced performance and consistently produced results across three distinct datasets. It was created as a response to the issue of models designed for a single dataset having a low generalization ability.

In order to accurately detect breast cancer and offer complete reasons for its predictions, a hybrid approach for breast cancer classification that integrates deep learning techniques was suggested by^27^. Convolutional neural networks (CNN) and the Light Gradient Boosting Model (LGBM) work together to exploit breast cancer. Numerous convolutional layers incorporate the model’s automated feature learning process, which LGBM then follows to generate class label predictions. This method emphasizes explainability but may be limited in capturing complex spatial dependencies.

### Problem statement

The reviewed literature shows notable improvements in the categorization of breast cancer utilizing both transformer-based designs and conventional CNNs. While hybrid methods like Deep ViT-CNN and ResMTUNet increase accuracy but struggle with high-resolution images and complicated tissue variability, shallow networks like ES-CNN offer computational cost but lack deep feature representation. Although they improve local and global feature extraction, dual-branch and attention-based models (AFFNet, SAWL-Net) frequently struggle with overfitting and computational issues. Although transformer-based techniques such as HFT-Net enhance interpretability and generalization, appropriate spatial–temporal representation is still limited. The development of the proposed BrCTransNet was driven by the need for an integrated framework that combines multiscale feature extraction, sequential spatial modeling, and optimized transformer-based classification to achieve high accuracy, robustness, and efficiency.

## Proposed methodology

**Fig. 1:**
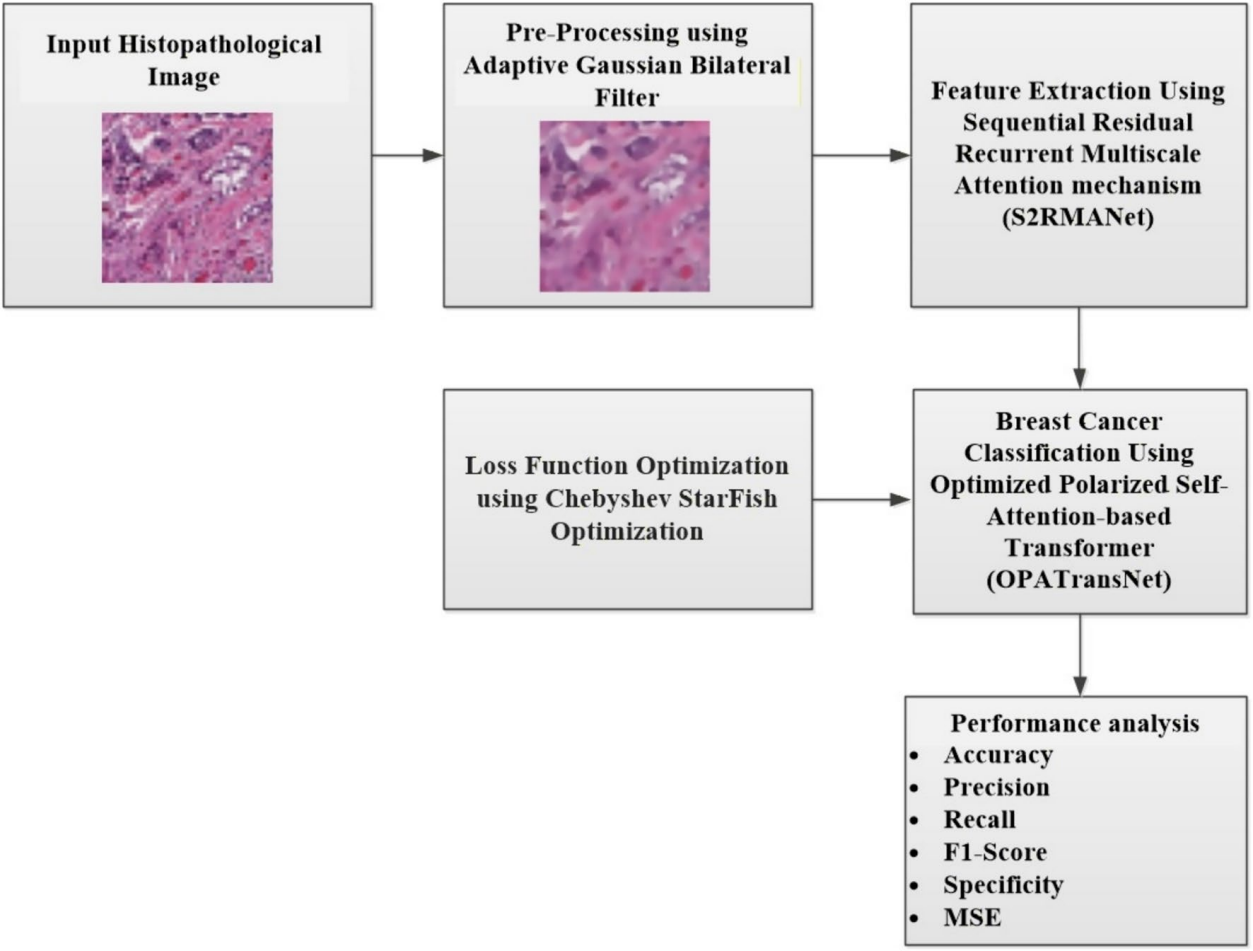
Workflow of proposed BrCTransNet model.

The proposed methodology for breast cancer classification begins with input medical images, which are first pre-processed using an AGB filter to effectively remove noise while preserving important edge and texture details crucial for accurate diagnosis. Following pre-processing, the images undergo feature extraction using an S2RMANet mechanism, which integrates Squeeze ResNet for spatial encoding, GRU for sequential spatial dependencies, and a multiscale attention block to capture varying granularities of visual information. The extracted features are then passed into a Transformer-based classification model, OPATransNet, where the conventional multi-head self-attention module is replaced with an Optimized Polarized Multi-Scale Self-Attention mechanism. The OPATransNet model decouples attention into spatial and channel branches, enhancing both local and global feature representations while improving computational efficiency. Finally, to ensure robust learning and fine-tuning, the entire model’s loss function is optimized using the ChStF algorithm, which leverages the global search capabilities of StarFish swarm behavior and the smooth convergence characteristics of Chebyshev mapping to avoid local minima and improve classification performance. The workflow of the proposed method is presented in Fig. 1.

### Data acquisition

The input for processing the proposed BrCTransNet is acquired from the publically available dataset^28^. The selected dataset consist of histological images, which are the gold standard for an accurate diagnosis of breast cancer. Histopathological images offer the cellular-level morphological information required for accurate identification between invasive ductal carcinoma (IDC) and non-IDC tissues, compared to mammography or ultrasound, which are mainly employed for screening and are constrained by tissue overlap and imaging errors. This dataset was selected to guarantee high diagnostic reliability and therapeutic relevance of the proposed model, since histological examination is ultimately the basis for clinical diagnosis and treatment planning.

### Adaptive Gaussian Bilateral filter

Conventional Gaussian blur filter employs a uniform smoothing effect across the entire image that makes the blurring edges and eliminating crucial diagnostic details. The smoothing may obscure subtle but important features. To address this limitation, the bilateral filter with adaptive Gaussian variant is designed. The adaptive Gaussian Bilateral (AGB) smooths the image by preserving edges and subtle boundaries that ensures the critical diagnostic features remain sharp and distinct. Besides, AGB filters adapt to the local characteristics of the image that provides context-aware smoothing that results in a more accurate and diagnostically valuable outcome. Let the input image be *K*, and the filtered output at pixel location *d* be $K_{D}^{a}$. Then:

1 $$K_{D}^{a} = \frac{1}{{z_{D} }}\sum\limits_{e \in \varphi } {M_{{\varepsilon_{h} }} } \left( {\left\| {D - E} \right\|} \right) \cdot M_{{\varepsilon_{m} }} \left( {\left| {K\left( D \right) - K\left( E \right)} \right|} \right) \cdot K\left( E \right)$$

where, *D* symbolizes the current pixel location, $e \in \varphi$ notates the neighboring pixels in a local window centered at *D*, $K\left( D \right)$ interprets the intensity of image at pixel *D*, $M_{{\varepsilon_{h} }}$ signifies the weights based on distance between *D* and *E* and $M_{{\varepsilon_{m} }}$ symbolizes the weights based on intensity difference. $z_{D}$ is the normalization factor to ensure weights sum to 1. Here, the parameters $M_{{\varepsilon_{h} }}$ and $M_{{\varepsilon_{m} }}$ are varied adaptively for enhancing the smoothing capability to improve the image quality.

### Feature extraction using S2RMANet

**Fig. 2:**
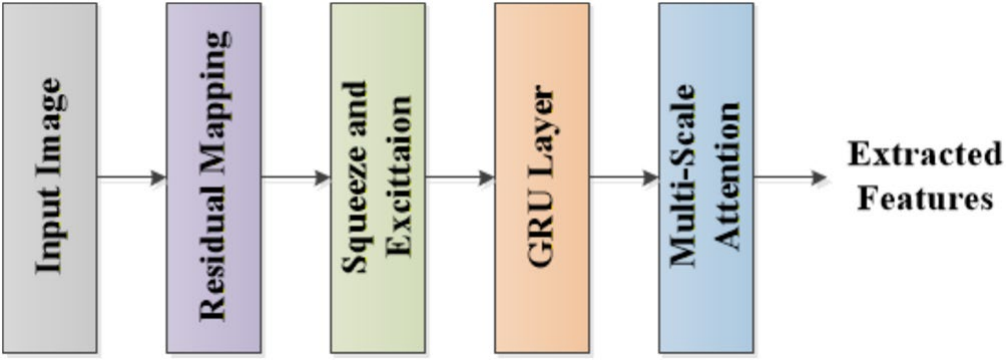
Structure of S2RMANet Model.

The proposed Sequential Residual Recurrent Multiscale Attention mechanism (S2RMANet) is utilized for extracting the sequential spatial dependencies and long-range contextual features. The S2RMANet integrates Squeeze ResNet, gated recurrent unit (GRU) and Multiscale Attention for obtaining efficient features for breast cancer classification. Here, the Squeeze ResNet component uses residual learning and parameter-efficient squeeze blocks to extract deep spatial features from histopathological images by preserving fine-grained texture and structure characteristics for identifying malignant patterns. The residual connections help mitigate vanishing gradients and allow the model to learn both shallow and deep features effectively. Besides, the consideration of GRU introduces sequential spatial modeling that allows the network to capture long-range contextual dependencies across spatial feature maps for capturing finer variations across tissue regions. The 2D spatial feature maps produced by the Squeeze ResNet backbone are first separated into non-overlapping patches (regions) in S2RMANet. A feature vector created by spatial pooling is used to represent each patch. Then, using a predetermined sequential ordering (row-wise, left-to-right, top-to-bottom), these patch-level feature vectors are flattened into a one-dimensional sequence. Sequential processing is made possible by this predictable ordering, which transforms the 2D spatial arrangement into an organized sequence. Also, the Multiscale Attention mechanism dynamically focuses on discriminative features at various spatial resolutions by assigning higher importance to informative channels and regions while suppressing irrelevant features by improving both robustness and interpretability of the classification. Thus, the proposed S2RMANet approach extracts the appropriate features for enhancing diagnostic accuracy in distinguishing benign from malignant cases. The structure of the proposed S2RMANet is presented in Fig. 2.

The Squeeze ResNet^29^ module combines ResNet’s residual learning with Squeeze-and-Excitation (SE) blocks for efficient spatial feature extraction. Convolutional blocks in the model are employed to extract local texture patterns, wherein the residual blocks enable deeper learning by mitigating vanishing gradients. SE blocks further enhance feature representation through channel-wise attention. Let the input image is represented as $K \in R^{Q \times M \times L}$, where *Q*, *M*, *L* are the height, width, and number of channels. The residual mapping in ResNet:

2 $$E_{rm} \left( K \right) = f\left( K \right) + K$$

where, $f\left( K \right)$ is the transformation that comprises of Convolution, batch normalization and ReLU activation function, and *K* is the residual connection.

The SE block compresses each feature map into a single representative value. It summarizes what each channel globally represents across the entire image.

3 $$b_{l} = \frac{1}{Q \times M}\sum\limits_{s = 1}^{Q} {\sum\limits_{m = 1}^{M} {K_{s,m,l} } } ,\,\,b \in R^{L}$$

The compressed values are passed through a lightweight neural network to learn features are most important. It captures complex dependencies between channels, re-weighting them based on relevance.

4 $$u = \delta \left( {\left( {Y_{2} } \right) \cdot \gamma \left( {Y_{1b} } \right)} \right) \in R^{L}$$

After identifying important channels, the SE block scales the original feature maps accordingly for enhancing meaningful ones and suppressing irrelevant ones. It fine-tunes the model’s focus and makes spatial features more discriminative for classification.

5 $$\tilde{K}_{s,m,l} = u_{l} .K_{s,m,l}$$

where, $Y_{1} \in R^{{\frac{L}{y} \times L}}$, $Y_{2} \in R^{{L \times \frac{L}{y}}}$ are weights, *γ* is sigmoid activation function and *y* is the reduction ratio. GRU is used to model sequential spatial dependencies across image patches or regions^30^. In order to facilitate contextual interaction between tissue regions, the GRU models processed a sequential series of flattened spatial feature patches created from 2D feature maps. It captures the continuity and interdependence between spatial regions. GRUs also maintain memory with fewer parameters that makes them efficient for processing large medical images. The GRU models sequential spatial dependencies across sequential features extracted from Squeeze ResNet outputs.

6 $$d_{r} = \delta \left( {S_{d} e_{r} + M_{d} o_{r - 1} } \right)$$

7 $$n_{r} = \delta \left( {S_{n} e_{r} + M_{n} o_{r - 1} } \right)$$

8 $$\hat{o}_{r} = \tanh \left( {\left( {S_{o} e_{r} } \right)M_{o} \left( {n_{r} \Theta \,o_{r - 1} } \right)} \right)$$

9 $$o_{r} = \left( {1 - d_{r} } \right)\Theta o_{r - 1} + d_{r} \Theta \,\hat{o}_{r}$$

where, $e_{r}$ symbolizes the input at time *r*, $o_{r}$ notates the hidden state at time *r*, $S_{ * }$ and $M_{ * }$ signifies the trainable weights, *Θ* interprets the element-wise multiplication, and *δ*, and *\tanh* are the hyperbolic tangent. Multi-scale attention mechanism module enhances discriminative representation by applying attention at multiple scales for focusing on important spatial regions. In this module, the features are pooled into multiple scales:

10 $$E = \left\{ {E_{1} ,E_{2} ,....E_{q} } \right\},\,\,E_{s} \in R^{{Q_{S} \times M_{S} \times L}}$$

Each $E_{s}$ is then up-sampled to a common size and concatenated. For each spatial location $\left( {s,m} \right)$, a weight is learned:

11 $$V_{s,m} = soft\max \left( {S_{z} * E_{s,m} + p_{z} } \right)$$

The multi-scale attention-weighted feature map is interpreted as:

12 $$E_{A} = \sum\limits_{s,m} {V_{s,m} } \cdot E_{s,m}$$

where, $S_{z}$, and $p_{z}$ are learnable parameters, and * defines the convolution operation. The proposed S2RMANet efficiently capturing deep hierarchical spatial, and long-range contextual features. Further enhancing the discriminability of these features, multiscale attention mechanisms selectively focus on relevant information across different resolutions. The extracted features are fed into the proposed OPATransNet model for disease classification.

### Breast cancer classification

**Fig. 3:**
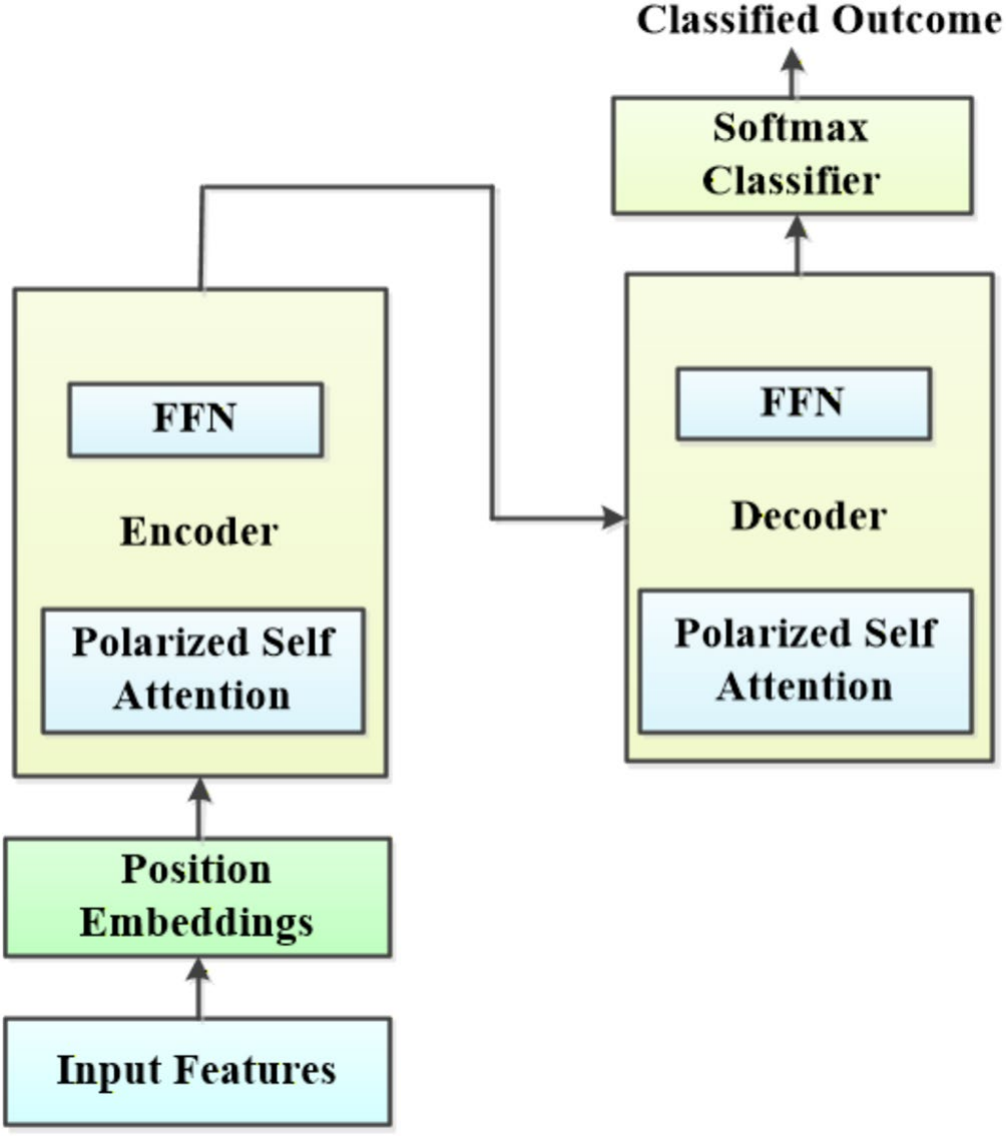
Structure of OPATransNet model.

Using the extracted features, the breast cancer classification is devised by the proposed Optimized Polarized Self-Attention-based Transformer (OPATransNet) model. The proposed OPATransNet model is designed with loss function optimization using the ChStF algorithm for enhancing the detection accuracy. In addition, the polarized self-attention mechanism is considered for modeling the multi-head attention mechanism for enhancing the performance through acquisition of multi-scale attention mechanism. The structure of proposed OPATransNet model for breast cancer detection is presented in Fig. 3.

**Patch embeddings**: The input features are converted into patches *A × A*, flattened, and projected to a token embedding space. Then, the positional embedding is employed for making the feature appropriate for processing through the transformer.

**Polarized self-attention**: The OPATransNet model improves feature representation through the learning of detailed, multi-scale spatial dependencies. It achieves this by integrating attention mechanisms that operate across various spatial scales. Furthermore, it combines query, key, and value representations with both global contextual information and element-wise enhancement. Initially, the polarized self-attention module processes the input feature map to generate a set of query vectors. For this, convolutional layer is used to transform the features into a more compact and query-specific representation. Then, it reorganizes the data to align with the expected attention format. It helps the model identify which spatial positions are relevant for further comparison. Thus the spatial query feature extraction from the input $E_{n}$ is interpreted as:

13 $$U_{f} = \infty_{x} \left( {Z_{x} \left( {Q_{x} \left( {E_{n} } \right)} \right)} \right)$$

where, $Q_{x}$ is the convolution to extract spatial query features, $Z_{x}$ is the permute and $\infty_{x}$ is devised to reshape to a query vector set. Then, the polarized self-attention module computes a set of key vectors that act as a reference for the queries. In this stage, compressing the spatial patterns across the entire image is employed to generate the single compact representation. The summary is generated through global average pooling and further processed through convolution and softmax to normalize the key values.

14 $$M_{f} = Y_{s} \left( {\infty_{x} \left( {Y_{g} \left( {Z_{m} \left( {\infty_{m}^{1} \left( {Q_{m} \left( {E_{n} } \right)} \right)} \right)} \right)} \right)} \right)$$

where, $Q_{m}$ is the convolution for spatial keys, $\infty_{m}^{1}$ notates the reshape operation before permutation, and $Z_{m}$ indicates the dimension permutation. $Y_{g}$ signifies the global mean pooling across spatial dimensions, $\infty_{m}^{2}$ signifies the reshape factor employed to match key format, and $Y_{s}$ is the softmax over key values that’s is used to generate the spatial attention distribution. Then, the attention scores are calculated between queries and global keys by comparing them. These scores indicate how much focus each spatial location should receive, based on how well its query matches the global key. The comparison results in a multi-dimensional attention map that highlights areas of interest across multiple spatial scales. These scores are then passed through a sigmoid function that squashes them into values between 0 and 1 for making them interpretable as gating signals:

15 $$W_{f} = Y_{a} \left( {\infty_{w} \left( {M_{f} \times U_{f} } \right)} \right)$$

where, $M_{f} \times U_{f}$ symbolizes the dot product between keys and queries gives attention score matrix, $\infty_{w}$ signifies the reshape factor employed to match spatial resolution, and $Y_{a}$ interprets the sigmoid activation. Then, the module calculates the value vectors. These are the parts of the input feature map that actually carry the content to be preserved or enhanced. Like the queries, the values are extracted using convolution, reshaped, and aligned with the structure of the attention scores. While the attention scores indicate where to focus, the value vectors tell us what information exists at each spatial position. It is interpreted as:

16 $$J_{f} = Z_{y} \left( {\infty_{y} \left( {Q_{y} \left( {E_{n} } \right)} \right)} \right)$$

where, $Q_{y}$ symbolizes the convolution for spatial value features, $\infty_{y}$ signifies the reshape factor employed to perform reshape operation and $Z_{y}$ symbolizes the permute to match attention mask format. Finally, the attention scores and value vectors are fused together by performing an element-wise multiplication. It signifies that each value vector is scaled by the corresponding attention score, enhancing or suppressing the features depending on their importance.

17 $$E_{n} = Q_{out} \left( {\infty_{out} \left( {Z_{out} \left( {J_{f} \Theta W_{f} } \right)} \right)} \right)$$

where, $J_{f} \Theta W_{f}$ is the spatial position that is enhanced by the corresponding attention score, $Z_{out}$ symbolizes the permute dimensions to original order, $\infty_{out}$ signifies the reshape factor, and $Q_{out}$ symbolizes the convolution to fuse the final enhanced spatial features. Here, the polarized self-attention significantly benefits in capturing long-range dependencies through the utilization of global keys that enables the model to understand relationships between distant elements in the input. Also, it facilitates the learning of multi-resolution spatial attention allows the model to focus on features at varying scales and granularities. By introducing element-wise modulation, it further enhances the precision of spatial features that results in more refined and informative representations. Thus, the polarized self-attention method preserves both spatial structure and contextual information to enhance the classification accuracy.

**Feed forward layer**: Each token is passed through a 2-layer Multi-Layer Perceptron (MLP):

18 $$FFN\left( d \right) = Q_{2} \cdot GeLU\left( {Q_{1d} + h_{1} } \right) + h_{2}$$

where, $Q_{1}$ and $Q_{2}$ are the weights, *GeLU* is the non-linear activation and the bias is signified as $h_{1}$, $h_{2}$.

**Layer normalization**: Normalization is applied before attention and FFN to stabilize training.

**Residual connection**: To prevent vanishing gradients and aid convergence, each sub-block has a skip connection: The residual connection ensures that gradients can flow backward easily and deeper layers can be trained efficiently.

**Loss function optimization**: The loss function optimization is devised based on the error function, which is optimized using the proposed ChStF algorithm. The error function is defined as:

19 $$N\left( C \right) = \frac{1}{z}\sum\limits_{c = 1}^{Z} {\left( {Y_{c} - \tilde{Y}_{c} } \right)}^{2}$$

where, the loss function (objective function) is interpreted as $N\left( C \right)$, the total solutions is indicated as *z*, the required solution is defined as $Y_{c}$ and the obtained solution is interpreted as $\tilde{Y}_{c}$.

**Chebyshev StarFish optimization algorithm**

The proposed Chebyshev StarFish Optimization (ChStF) Algorithm includes the Chaotic Chebyshev Mapping in the conventional StarFish optimization algorithm^31^ for enhancing the diversification phase to solve the issue of local optimal trapping and convergence rate. ChStF is used to tune OPATransNet hyperparameters during training, by optimizing loss. In particular, ChStF avoids local minima, speeds up convergence, and enhances generalization performance by adaptively fine-tuning the models hyperparameter by reducing the classification error. In compared to traditional gradient-based optimization alone, the integration of chaotic Chebyshev mapping improves population diversity in the early stages, and the StarFish-based intensification refines solutions close to optimal regions, leading to faster and more stable learning.

**Initialization**: To randomly distribute the population of StarFish (candidate solutions) across the search space. Let the population size be *G*, and the number of design variables (problem dimension) be *B*. The position matrix $C \in R^{G \times B}$ stores all candidate solutions:

20 $$C = \left[ {\begin{array}{*{20}c} {C_{1,1} } & \cdots & {C_{1,B} } \\ \vdots & \ddots & \vdots \\ {C_{G,1} } & \cdots & {C_{G,B} } \\ \end{array} } \right]$$

Each position is initialized as:

21 $$C_{k,v} = n{}_{v} + w \cdot \left( {x_{v} - n_{v} } \right)\,,\,\,\,\,k = 1,...G;\,\,v = 1,...B$$

where, $C_{k,v}$ signifies the $v^{th}$ dimension of the $k^{th}$ StarFish and *w* notates the random number and $n_{v}$, and $x_{v}$ signifies the lower and upper bounds for the $v^{th}$ variable. The fitness vector is computed using the objective function $N\left( C \right)$ based on error, which is presented in Eq. (19).

**Diversification**: The StarFish simulates using all five of its arms to explore the surrounding environment. In this phase, five dimensions are randomly selected for each StarFish to update its position toward the best-known solution. The diversification balances global exploration with partial learning from the most successful candidates. The movement is guided using a dynamic step influenced by cosine and sine functions to simulate natural and flexible arm movement in StarFish. Two distinct strategies are applied based on the problem dimensionality:

(i) Multidimensional Search Pattern is devised for the condition *B > 5*. StarFish arms with eyes at the tips explore five randomly chosen dimensions out of the total *B*.The update equation for the selected dimensions $v \in w^{5}$ is:
  22 $$A_{k,v}^{s} = \left\{ {\begin{array}{*{20}c} {C_{k,v}^{s} + \gamma_{1} \left( {C_{good,v}^{s} - C_{k,v}^{s} } \right)\cos \,d,} & {w \le 0.5} \\ {C_{k,v}^{s} + \gamma_{1} \left( {C_{good,v}^{s} - C_{k,v}^{s} } \right)\sin \,d,} & {w > 0.5} \\ \end{array} } \right.$$
  where, $C_{k,v}^{s}$ signifies the current position in selected dimensions, $C_{good,v}^{s}$ notates the best solution in those dimensions, $w\sim P\left( {0,1} \right)$ indicates the random number and $\gamma_{1} = \left( {2w - 1} \right)^{v}$ symbolizes the exploration amplitude factor. $d = \frac{\pi }{2} \cdot \frac{s}{{s_{\max } }}$ indicates the orientation twist over iterations, *s* signifies the current iteration, $s_{\max }$ symbolizes the max iterations. If a new position is out-of-bounds, it is reverted:
  23 $$C_{k,v}^{s + 1} = \left\{ {\begin{array}{*{20}c} {A_{k,v}^{s} } & {if\,within\,bounds} \\ {C_{k,v}^{s} } & {otherwise} \\ \end{array} } \right.$$
(ii) Uni-dimensional Search Pattern is devised for the condition *B łe 5*. StarFish explore along a single randomly selected dimension, inspired by the motion of one active arm.
  24 $$A_{k,r}^{s} = D_{s} C_{k,r}^{s} + H_{1} \left( {C_{g1,r}^{s} - C_{k,r}^{s} } \right) + H_{2} \left( {C_{g2,r}^{s} - C_{k,r}^{s} } \right)$$
  where, *v* symbolizes the randomly selected dimension, $C_{g1,r}^{s}$, $C_{g2,r}^{s}$ signifies the positions from two random StarFish, $H_{1} H_{2} \sim P\left( { - 1,1} \right)$ indicates the directional scalars, and $D_{s} = \frac{{s_{\max } - s}}{{s_{\max } }}$ defines the energy of the StarFish that reduces over time. Here, diversification ensures localized movement when the search space is small. Here, Chebyshev chaotic mapping is used to initialize or perturb the StarFish positions. It helps the algorithm explore the search space more thoroughly. The Chebyshev map is a deterministic chaotic function defined as:
  25 $$A_{k,r}^{s} = \cos \left( {E \cdot A_{k,r}^{s - 1} } \right)$$
  where, the control factor is defined as *E*. Here, the adaptive scaling of the control factor helps to balance diversification and intensification to avoid getting stuck in local optima. Also, the control factor adjusts the step size or influence of chaotic perturbations over iterations and enhances the convergence rate. The novel solution update after incorporating the Chebyshev mapping is expressed as:
  26 $$A_{k,r}^{s} = 0.5\,\left[ {\cos \left( {E \cdot A_{k,r}^{s - 1} } \right)} \right] + 0.5\left[ {D_{s} C_{k,r}^{s} + H_{1} \left( {C_{g1,r}^{s} - C_{k,r}^{s} } \right) + H_{2} \left( {C_{g2,r}^{s} - C_{k,r}^{s} } \right)} \right]$$

Thus, the solution update with Chebyshev mapping ensures non-repetitive, ergodic, and unpredictable diversification that is significant in preventing premature convergence.

**Intensification**: To refine search results and intensify search near promising areas, using preying and regeneration mechanisms. Preying behavior is employed through parallel two-directional search that simulates movement toward or away from the best position using distance vectors. To simulate the StarFish’s food-hunting instincts, the preying behavior moves StarFish toward or away from the best-known solution using a two-directional strategy. Each StarFish considers multiple candidate directions and randomly selects two promising ones to explore in parallel. The process promotes both convergence and diversity, as some StarFish move closer to food while others retreat to avoid predators. It mimics real-world animal behavior where movement is based on both attraction and evasion. Initially, compute five distance vectors to find the best position:

27 $$p_{a} = C_{good}^{s} - C_{av}^{s} ,\,\,\,a = 1,2,...5$$

where *av* is the index of a randomly selected StarFish. Then, the position update is explained as:

28 $$A_{k}^{s} = C_{k}^{s} + w_{1} p_{a1} + w_{2} p_{a2}$$

where, $w_{1} \sim \left( {0,1} \right)$, $p_{a1}$, $p_{a2}$ are random distance vectors among the five. It allows both advancing and retreating movement to improve diversification-intensification balance. The last StarFish in the population undergoes a regeneration process, simulating biological arm regeneration that occurs slowly and independently. This step ensures that even the least fit individual has a mechanism to re-enter the competition by updating its position more gradually. It prevents premature exclusion and enhances diversity over time. The regeneration mimics how real StarFish survive by rebuilding from damage, adding a slow but critical mechanism for escaping stagnation. Here, only the last StarFish *k = G* performs this action to simulate arm regeneration under stress.

29 $$A_{G}^{s} = \exp \left( {\frac{s - G}{{s_{\max } }}} \right) \cdot C_{G}^{s}$$

As $s \to s_{\max }$, the regeneration influence increases and encourages new directions for convergence. Then, the boundary constraint for intensification phase is devised to check if a position is outside the search space:

30 $$C_{k,v}^{s + 1} = \left\{ {\begin{array}{*{20}c} {A_{k}^{s} } & {if\,n_{u} \le A_{k}^{s} \le x_{u} \,} \\ {n_{u} } & {if\,A_{k}^{s} < n_{u} \,} \\ {x_{u} } & {if\,A_{k}^{s} > x\,} \\ \end{array} } \right.$$

The boundary constraint ensures all solutions are kept valid within search boundaries. The pseudo-code of the proposed ChStF algorithm is presented in Algorithm 1.

**Algorithm 1:**
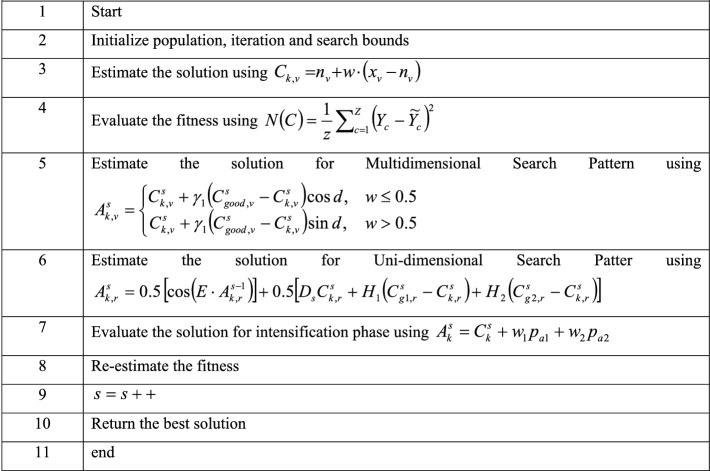
Pseudo-code for ChStF Algorithm.

Thus, using the solution accomplished by the ChStF algorithm, the loss function optimization is devised.

## Result and discussion

The proposed BrCTransNet model for breast cancer detection is implemented in PYTHON and is assessed based on various measures using Histopathology images. The Proposed method was analysed and outperforms traditional ResMTUNet^23^, Deep ViT-CNN^22^, ES-CNN^21^ and SAWL-Net^25^ models to demonstrate its effectiveness.

**Dataset source:**

The experimental evaluation is conducted using the publicly available Breast Histopathology Images (BreakHis) dataset, obtained from Kaggle^28^. The dataset consists of hematoxylin and eosin (H&E) stained breast tissue images collected from multiple patients at 40 × magnification. The dataset includes two primary categories: IDC (Class 0) and non-IDC (Class 1), exhibiting high inter-patient and intra-class variability.

**Input representation and patch extraction:**

Instead of processing whole-slide images directly, a patch-based learning strategy is employed to effectively capture localized morphological patterns. Although the dataset comprises 162 breast cancer slide images. Each slide image is divided into non-overlapping patches of size 224 × 224 pixels, yielding an average of 25–30 patches per image. Consequently, this results in approximately 4,000–4,800 patches in total, which are used for model training and evaluation and are adequate for deep transformer-based learning. Patches dominated by background or containing insufficient tissue information are excluded. The extracted patches are normalized and preprocessed using the AGB filter prior to feature extraction.

**Patient-wise data partitioning:**

To ensure unbiased evaluation and prevent data leakage, patient-wise data partitioning is strictly enforced. All patches originating from a single patient are assigned exclusively to either the training, validation, or testing set. The dataset is split at the patient level into 70% training, 15% validation, and 15% testing sets. This protocol ensures that the model is evaluated on completely unseen patient data and better reflects real-world clinical deployment conditions.

Table 1 shows the training and reproducibility details. Table 2 shows the hyperparameter setting. The OPATransNet model is trained using the Adam optimizer with an initial learning rate of 0.0001. The batch size is set to 16, and the model is trained for 200 epochs. Categorical cross-entropy is employed as the loss function. Dropout with a rate of 0.5 is applied to reduce overfitting. The ChStF algorithm is used as a meta-optimization strategy to fine-tune loss-related learning parameters. The population size is set to 30 StarFish agents, and the maximum number of iterations is fixed at 50. The Chebyshev control parameter is set to 4 to induce chaotic behavior for enhanced exploration. During the diversification phase, five dimensions are randomly selected for position updates. The search space boundaries are normalized within the range [0, 1].

**Table 1:** Training and Reproducibility Details.

Epochs	200
Device name	DESKTOP-UA8M7M2
Processor	Intel(R) Core(TM) i5-5200U CPU @ 2.20 GHz 2.20 GHz
Installed RAM	4.00 GB (3.88 GB usable)
Device ID	CAFC40C0-38BA-491B-AD23-328A236CC32A
Product ID	00330-80000-00000-AA293
System type	64-bit operating system, × 64-based processor
Pen and touch	No pen or touch input is available for this display

**Table 2:** Hyperparameter Setting.

Parameter	Value
OPATransNet	
Optimizer	Adam
Initial learning rate	0.0001
Batch size	16
Epochs	200
Loss function	Categorical Cross-Entropy
Dropout rate	0.5
ChStF	
Population size	30
Max iterations	50
Chebyshev control factor	4
Exploration amplitude	0.5
Dimensional selection	5 dimensions
Search bounds (LB, UB)	[0,1]

**Fig. 4:**
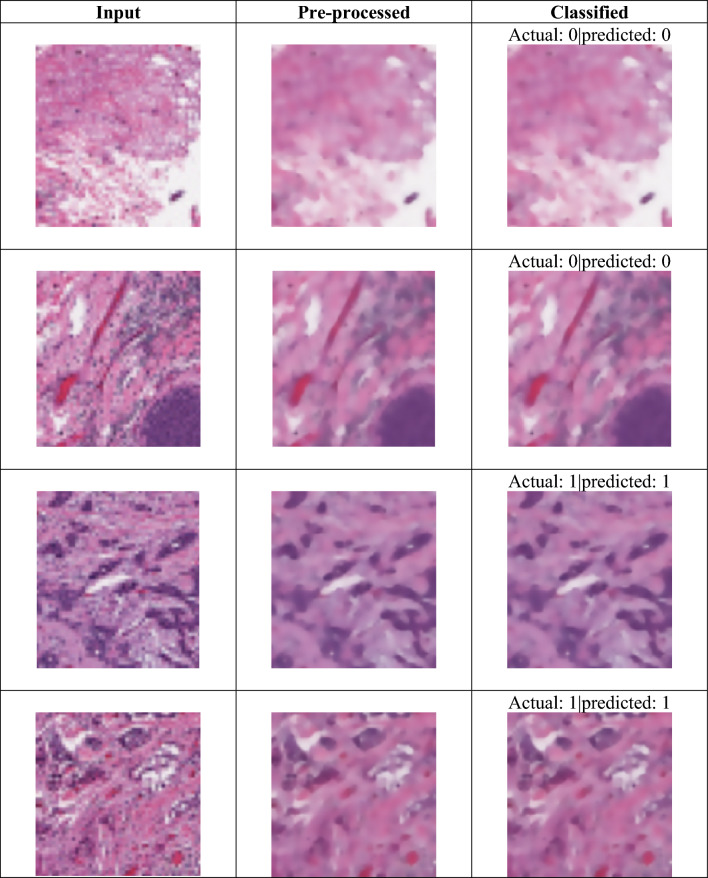
Experimental Outcome of BrCTransNet.

**Experimental outcome**: The experimental outcome of the proposed BrCTransNet along with the pre-processed outcome is presented in Fig. 4.

**Fig. 5:**
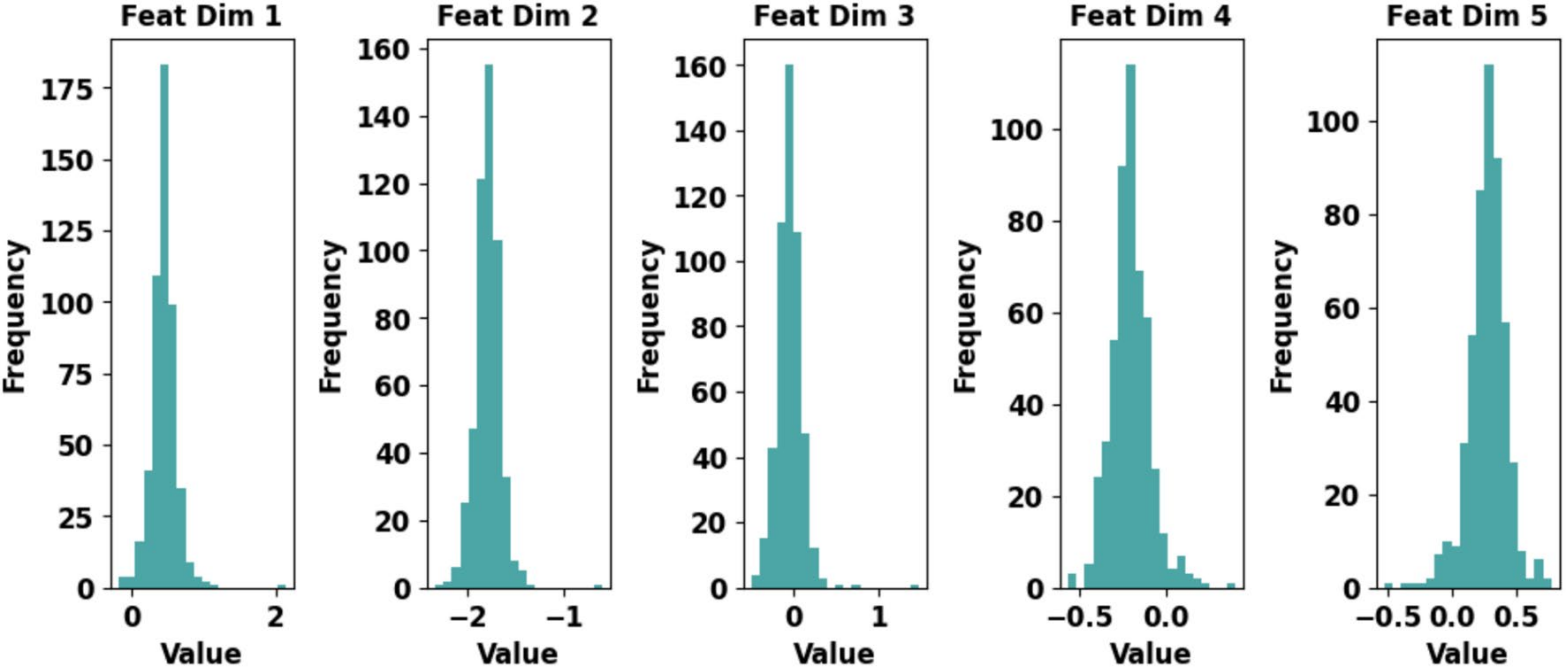
Analysis of Feature Extraction.

**Analysis of feature extraction**: Figure 5 shows the distribution of selected feature dimensions extracted from the final S2RMANet layer after Batch Normalization. These histograms demonstrate a balanced and symmetric spread of normalized feature activations across dimensions, indicating stable feature scaling and reduced bias among feature channels. It should be noted that the purpose of this analysis is not to imply strict Gaussian behavior, but to highlight the consistency and normalization of learned feature representations, which contribute to improved convergence and generalization in the subsequent transformer-based classification stage.

**Fig. 6:**
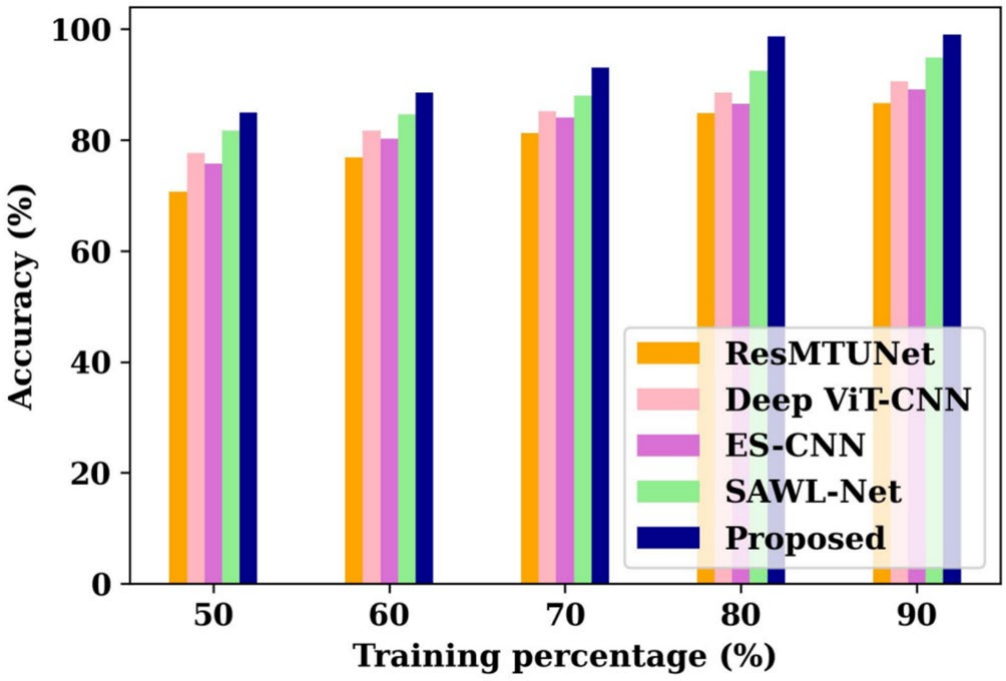
Analysis based on Accuracy.

Accuracy presented in Fig. 6 with its detailed analysis in Table 3 measures the ratio of correctly classified instances to the total number of instances. Here, the AGB filter improves image clarity by reducing noise while retaining edge structures that makes feature extraction more precise. Besides, the S2RMANet mechanism captures multi-level spatial dependencies and long-range contextual relationships across tissue regions. The OPATransNet provides enhanced contextual understanding across both spatial and channel dimensions that allow the Transformer to better distinguish between malignant and benign regions. Also, the ChStF for fine-tuning weights leads to highly accurate predictions.

**Table 3:** Analysis based on Accuracy.

Training percentage	ResMTUNet	Deep ViT-CNN	ES-CNN	SAWL-Net	Proposed
50	70.64	77.67	75.71	81.64	84.94
60	76.89	81.73	80.21	84.60	88.56
70	81.17	85.13	84.04	88.00	93.05
80	84.79	88.53	86.56	92.49	98.65
90	86.65	90.61	89.09	94.80	98.97

**Fig. 7:**
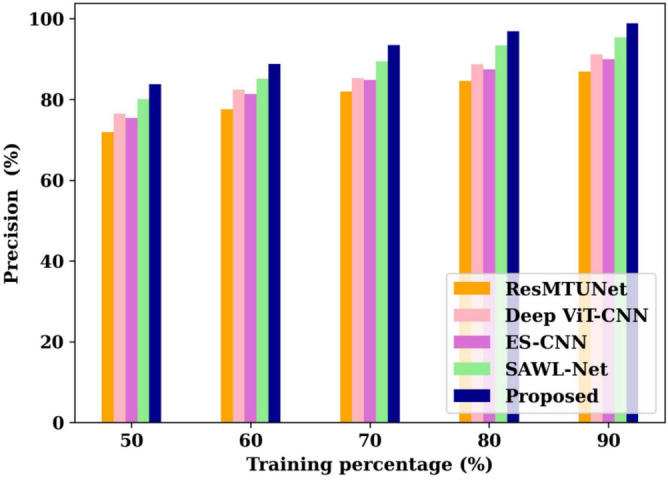
Analysis based on Precision.

Figure 7 illustrates the precision analysis with its detailed outcome in Table 4. Here, OPATransNet reduces false positives by focusing attention only on the most relevant spatial and channel areas using decoupled attention mechanisms. Besides, the multiscale attention captures finer variations in lesion texture and boundaries for reducing misclassifications. The proposed BrCTransNet model is better at identifying true malignancies due to the detailed spatial relationships and enhanced semantic representations.

**Table 4:** Analysis based on Precision.

Training percentage	ResMTUNet	Deep ViT-CNN	ES-CNN	SAWL-Net	Proposed
50	71.89	76.53	75.42	80.06	83.81
60	77.58	82.45	81.33	85.09	88.84
70	81.95	85.27	84.82	89.46	93.44
80	84.55	88.75	87.42	93.39	96.92
90	86.93	91.13	90.02	95.32	98.86

**Fig. 8:**
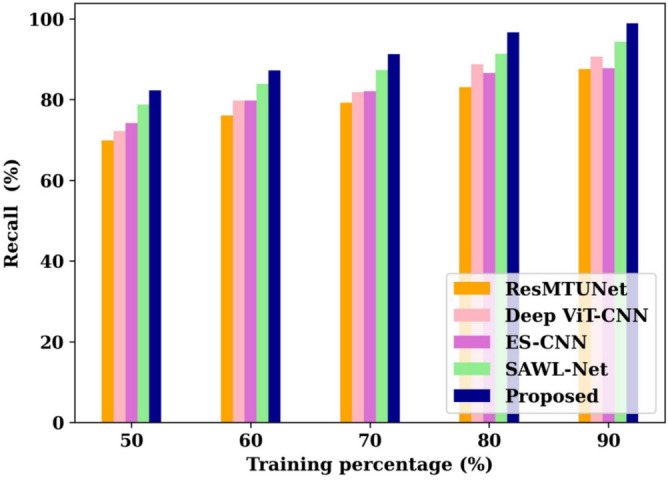
Analysis based on Recall.

Figure 8 illustrates the recall analysis with its detailed outcome in Table 5. Here, S2RMANet considers the recurrent component that ensures the significant feature context is retained across regions, which helps the model detect small or ambiguous cancerous features. The OPATransNet ensures global context is preserved, reducing missed detections. The proposed BrCTransNet model demonstrates the high recall that reflects its robustness in identifying all possible malignancies, even in complex images.

**Table 5:** Analysis based on Recall.

Training percentage	ResMTUNet	Deep ViT-CNN	ES-CNN	SAWL-Net	Proposed
50	69.84	72.24	74.20	78.79	82.29
60	76.08	79.80	79.79	83.93	87.21
70	79.26	81.88	82.08	87.33	91.26
80	83.09	88.78	86.58	91.38	96.66
90	87.59	90.64	87.78	94.37	98.91

**Fig. 9:**
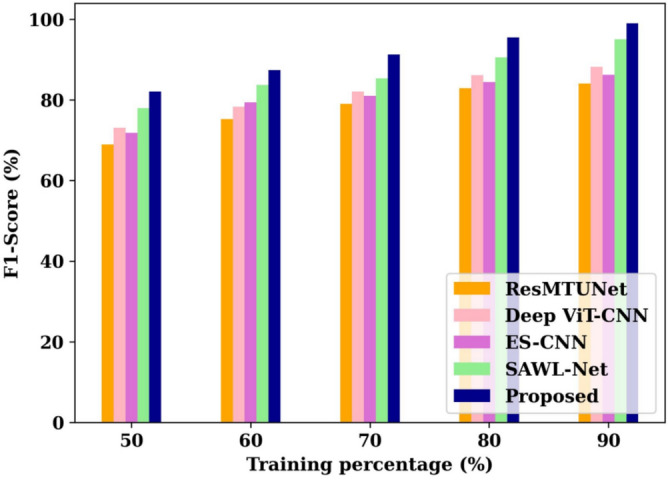
Analysis based on F1-Score.

Figure 9 illustrates the F1-Score analysis with its detailed outcome in Table 6. Here, the balanced architecture of the proposed BrCTransNet model ensures the enhanced F1-score that demonstrates the harmonic mean. OPATransNet avoids the usual trade-off seen in other models between precision and recall by optimizing attention on both local and global structures.

**Table 6:** Analysis based on F1-score.

Training percentage	ResMTUNet	Deep ViT-CNN	ES-CNN	SAWL-Net	Proposed
50	68.96	73.12	71.79	77.91	82.06
60	75.21	78.26	79.35	83.72	87.43
70	79.04	82.10	80.99	85.36	91.26
80	82.87	86.15	84.39	90.51	95.54
90	84.08	88.23	86.25	95.00	98.98

**Fig. 10:**
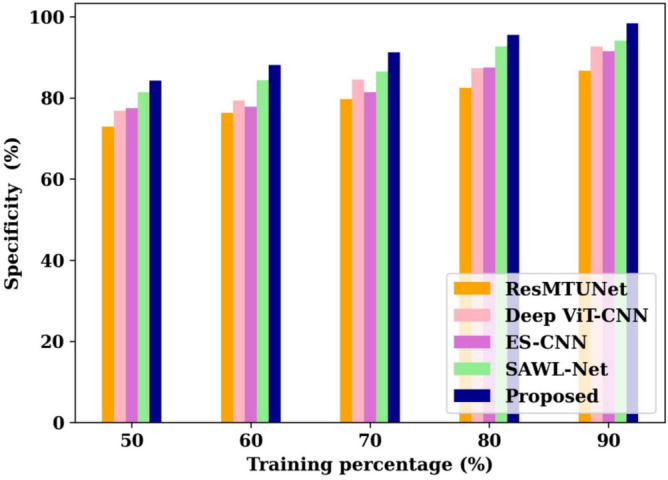
Analysis based on Specificity.

Figure 10 illustrates the Specificity analysis with its detailed outcome in Table 7. The pre-processing step using the AGB filter significantly cleans up benign tissue noise, preventing false positives. Besides, OPATransNet with selective focus on discriminative areas allows the model to effectively ignore non-pathological regions, improving specificity.

**Table 7:** Analysis based on specificity.

Training percentage	ResMTUNet	Deep ViT-CNN	ES-CNN	SAWL-Net	Proposed
50	72.91	76.84	77.48	81.42	84.25
60	76.30	79.36	77.81	84.37	88.09
70	79.70	84.50	81.43	86.46	91.26
80	82.44	87.24	87.45	92.70	95.53
90	86.71	92.61	91.51	94.12	98.36

**Fig. 11:**
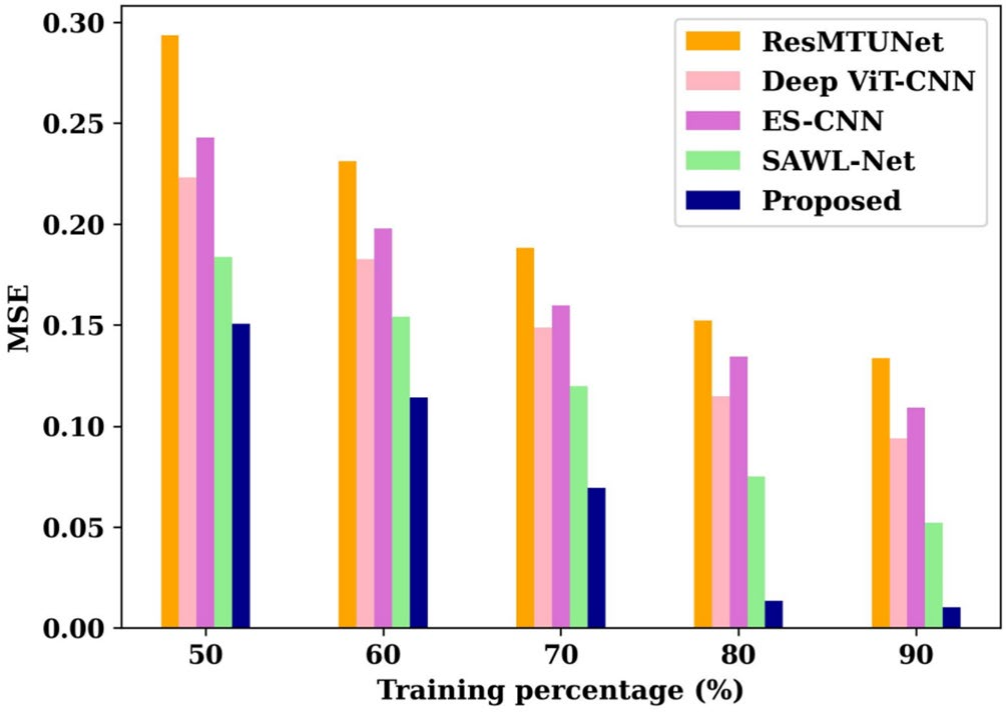
Analysis based on MSE.

Figure 11 illustrates the MSE analysis with its detailed outcome in Table 8. The ChStF algorithm tunes the loss landscape to achieve smoother convergence, minimizing prediction error. The learning process avoids overfitting or underfitting due to the combination of Chebyshev mapping based diversification and StarFish swarm dynamics based on adaptive intensification.

**Table 8:** Analysis based on MSE.

Training percentage	ResMTUNet	Deep ViT-CNN	ES-CNN	SAWL-Net	Proposed
50	0.2936	0.2233	0.2429	0.1836	0.1506
60	0.2311	0.1827	0.1979	0.1540	0.1144
70	0.1883	0.1487	0.1596	0.1200	0.0695
80	0.1521	0.1147	0.1344	0.0751	0.0135
90	0.1335	0.0939	0.1091	0.0520	0.0103

**Fig. 12:**
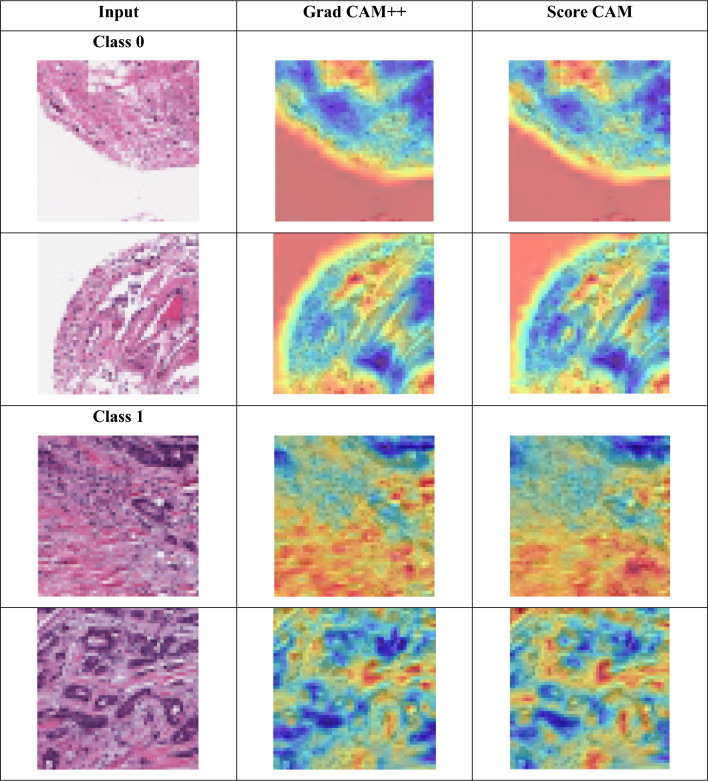
Visualizations of Grad-CAM +  + and Score-CAM for Class 0 and Class 1.

Grad-CAM +  + and Score-CAM visualization results for Class 0 and Class 1 are shown in Fig. 12, illustrating the interpretability of the proposed BrCTransNet model. These visualizations focus attention to the areas that have a major effect on the model’s classification outcomes. Limited aberrant tissue portions are shown by the sparse and isolated highlighted patches for Class 0. Class 1 activation maps, in contrast, highlight aberrant morphological features and exhibit broad and intense attention in dense cellular areas. While Score-CAM provides highly interpretable coverage of a wider spatial context, Grad-CAM +  + maps offer finer-grained attention on important tissue boundaries. Overall, the proposed approach efficiently learns and attends to diagnostically important regions, guaranteeing accurate and comprehensible classification of breast cancer.

**Fig. 13:**
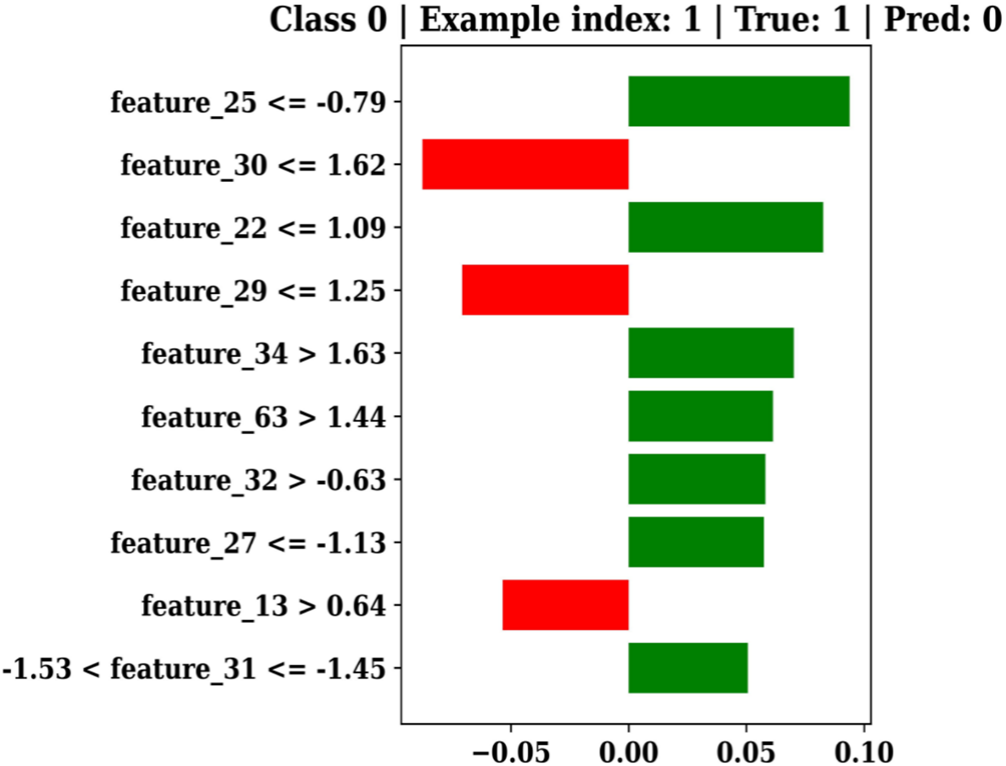
LIME-Based Interpretation of Model Prediction for Class 0.

The model’s prediction for Class 0 is interpreted using LIME, as shown in the Fig. 13. It illustrates how distinct characteristics influenced the ultimate choice. Red bars show features that were against the forecast, and green bars show those that were in support of Class 0. In contrast to features_62 and_60, which somewhat contradict the benign classification, features_26,_25, and_47 significantly support it. The prediction accurately matches the true label (True: 0, Pred: 0), demonstrating that the model’s assessment is comprehensible and based on pertinent localized characteristics taken from the histopathology data.

**Fig. 14:**
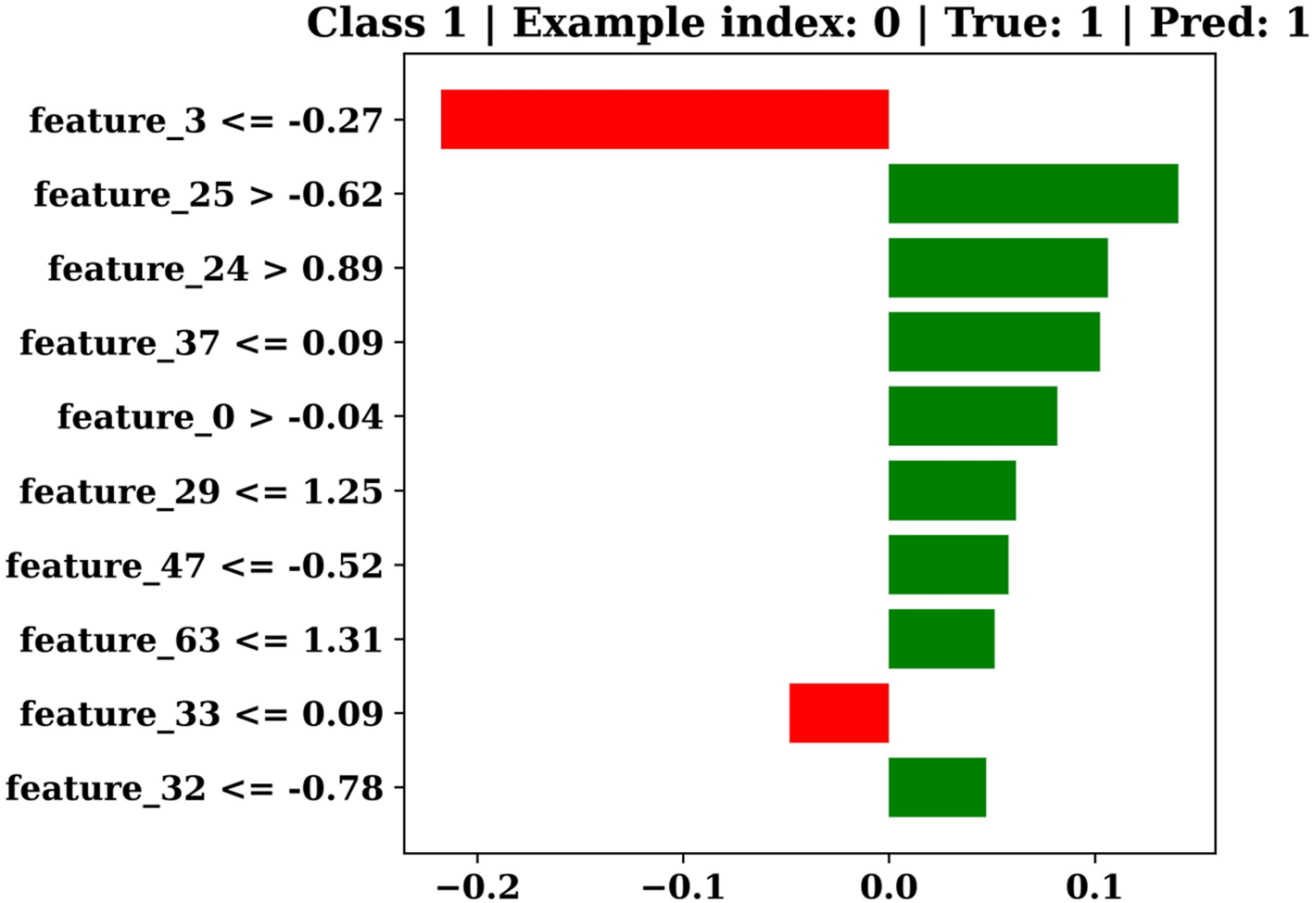
LIME-Based Interpretation of Model Prediction for Class 1.

The LIME-based interpretation of the model’s prediction for Class 1 (malignant) is shown in Fig. 14. While the red bars show features that contradict the prediction, the green bars show those that favourably impacted the model’s selection for Class 1. While features_57 and feature_26 had a negative contribution, features_11, feature_31, feature_10, and feature_8 significantly supported the malignant classification. The model accurately classified the sample as Class 1 (True: 1, Pred: 1), indicating that significant and physiologically relevant factors influenced the decision. This validates the interpretability and dependability of the proposed BrCTransNet model.

**Fig. 15:**
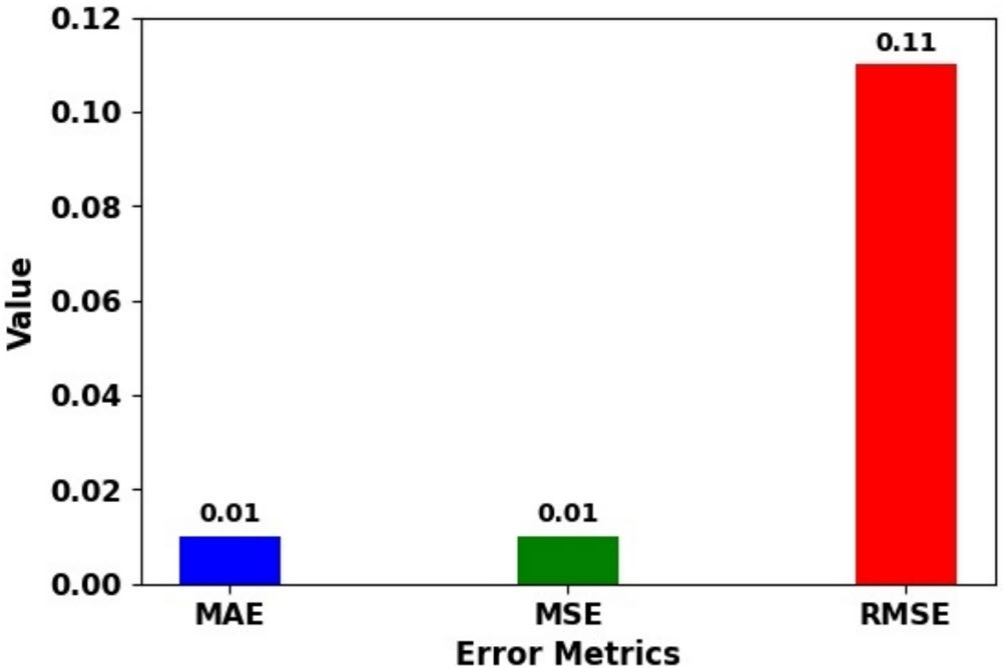
Comparison of Error Metrics.

**Fig. 16:**
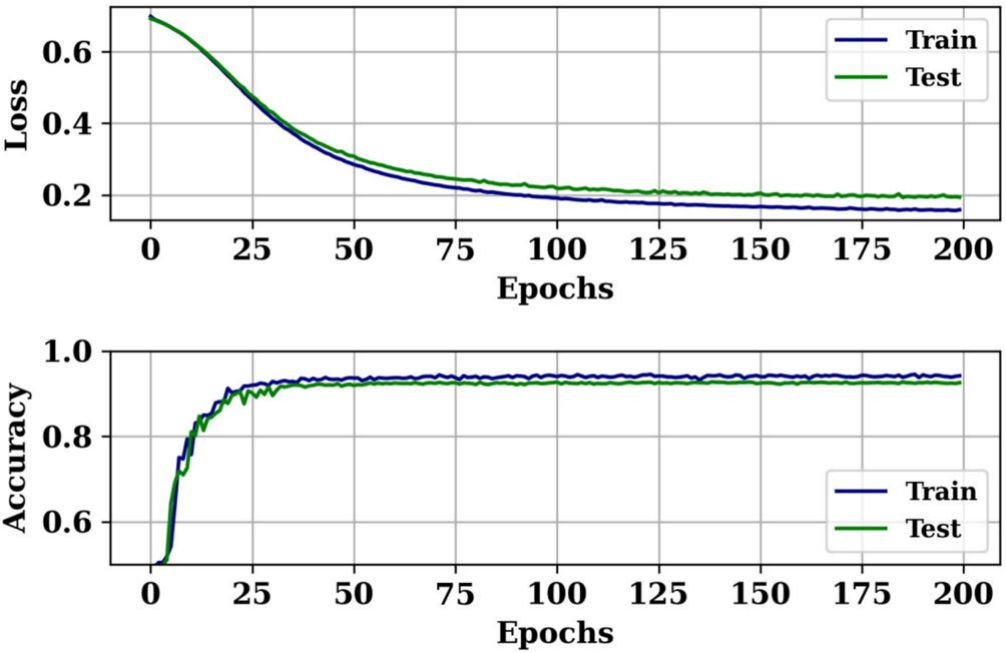
Analysis based on Accuracy-Loss.

Figure 15 shows the comparison of error metrics. The model’s prediction errors are small and continuously around the true values, as indicated by the observed Mean Absolute Error (MAE) and Mean Squared Error (MSE) values of 0.01. The low Root Mean Squared Error (RMSE) value of 0.11 indicates that there are rarely significant differences between expected and actual results. Overall, these results validate that the proposed model achieves high classification precision and stability, demonstrating its robust learning capacity and efficient ChStF algorithm improvement. Figure 16 illustrates the accuracy-loss analysis, The proposed BrCTransNet model demonstrates a faster convergence to high accuracy and low loss due to ChStF ability to escape local minima early in training. Besides, OPATransNet enables efficient representation learning that leads to fewer training iterations needed to achieve optimal performance.

**Fig. 17:**
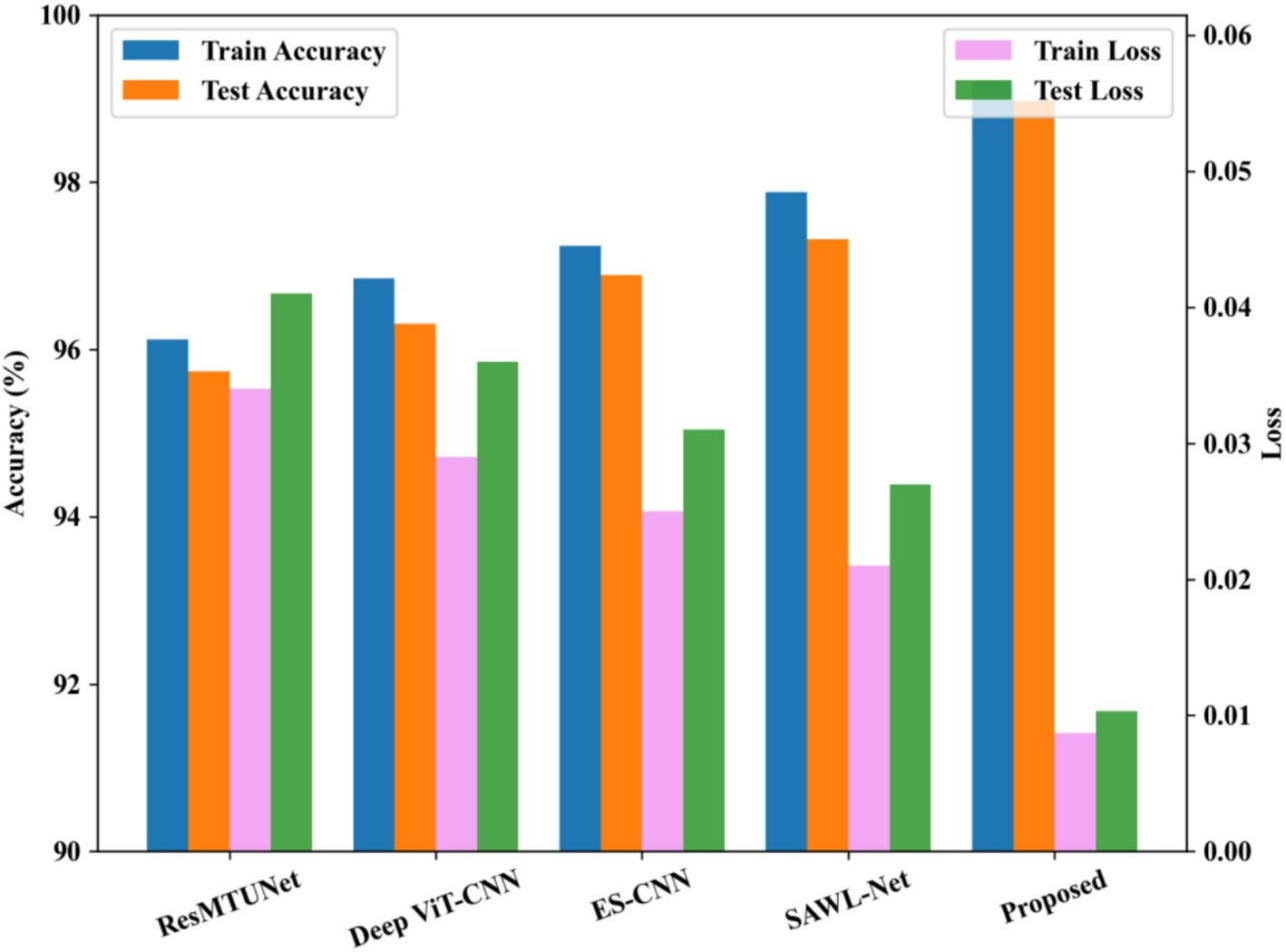
Comparison of Training and Testing Accuracy and Loss for Breast Cancer Classification Models.

Figure 17 shows the comparison of training and testing accuracy and loss for breast cancer classification models. According to the analysis, the proposed BrCTransNet maintains the lowest training and testing loss values of roughly 0.01 while achieving the best performance with training accuracy near 99% and testing accuracy around 98.9%. By contrast, SAWL Net records training accuracy of about 97.9% and testing accuracy of about 97.3%, with higher loss values of about 0.025–0.03. BrCTransNet provides better convergence and generalization performance than ES-CNN, Deep ViT CNN, and ResMTUNet, which exhibit lower accuracies between 96 and 97% and larger losses between 0.03 and 0.045.

**Fig. 18:**
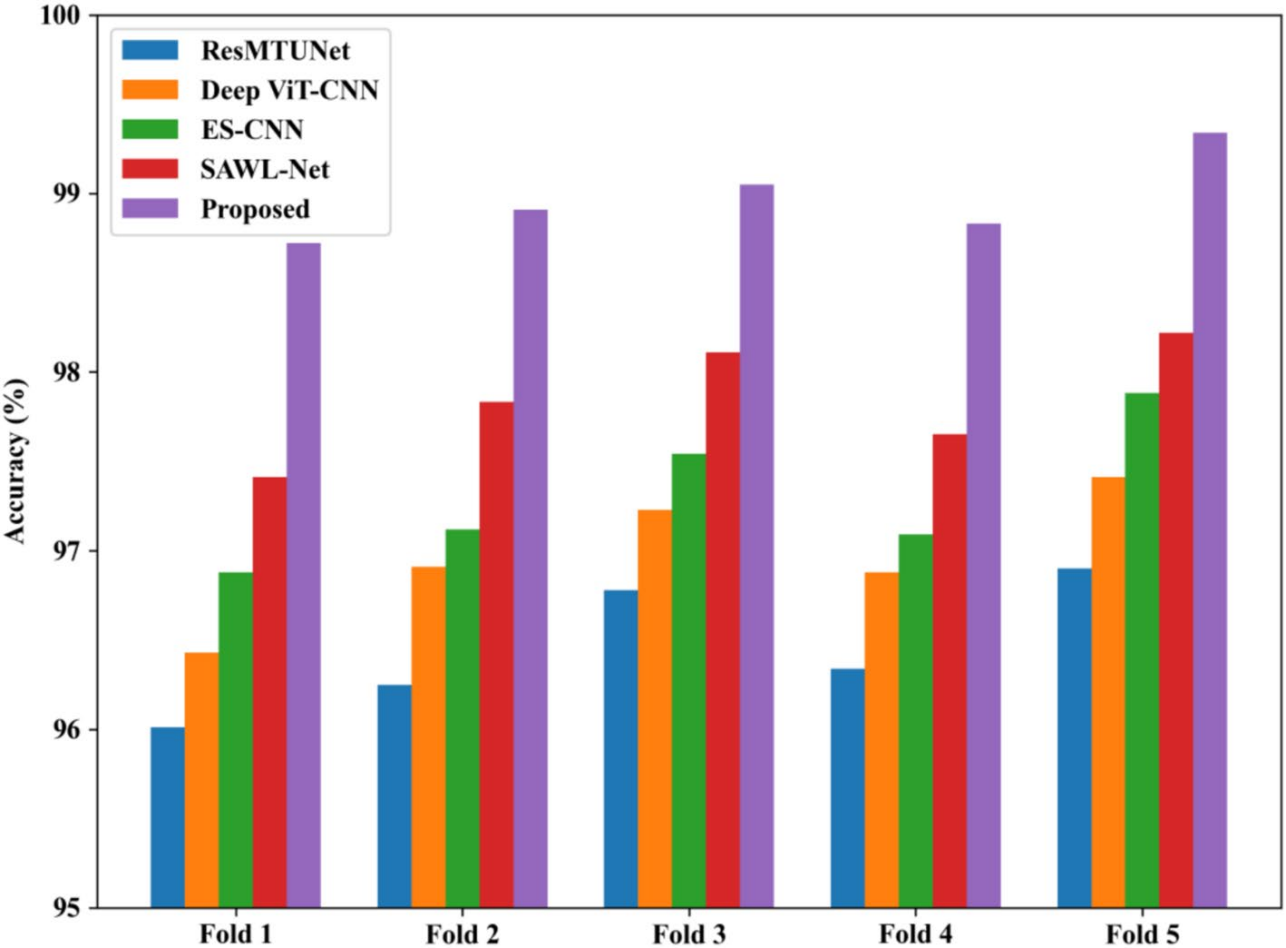
Analysis of cross-validation.

The cross-validation accuracy comparison for several models across five folds is shown in Fig. 18. With scores of roughly 98.7%, 98.9%, 99.1%, 98.8%, and 99.3% for Folds 1 through 5, respectively, the proposed BrCTransNet consistently obtains the highest accuracy in all folds. In contrast, SAWL-Net achieves accuracies between 97.4 and 98.2%, ES-CNN between 96.9 and 97.9%, Deep ViT-CNN between 96.4 and 97.4%, and ResMTUNet between 96.0 and 96.9%. These findings show that BrCTransNet has better stability and generalization capabilities across various data splits.

**Fig. 19:**
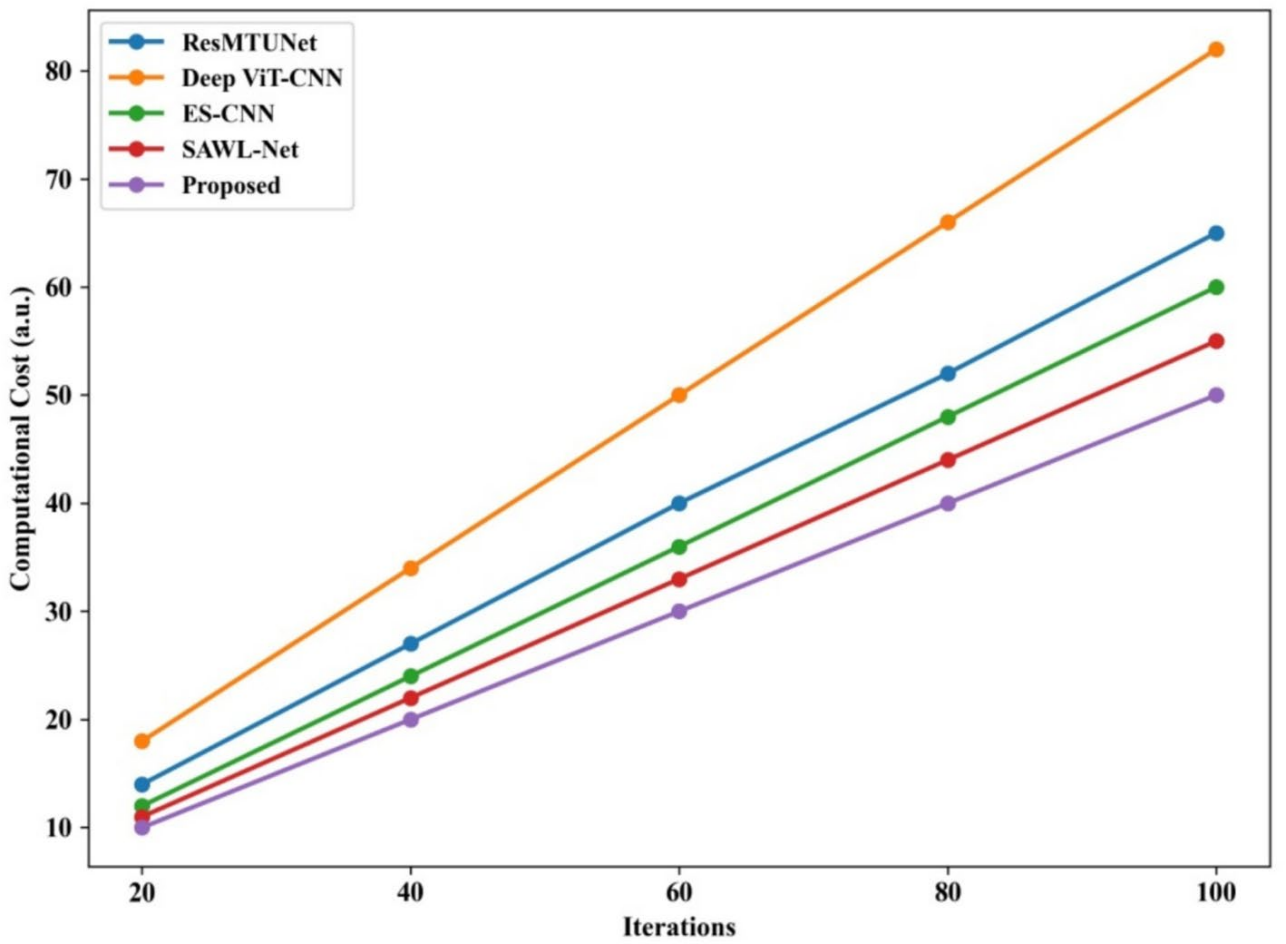
Analysis of Computational Cost.

Figure 19 shows the analysis of computational cost. The models computational costs at 100 iterations are as follows: Deep ViT-CNN is 82 a.u., ResMTUNet is 65 a.u., ES-CNN is 60 a.u., SAWL-Net is 55 a.u., and the proposed method is 50 a.u. According to the figure, Deep ViT-CNN needs the most resources, whereas the proposed method achieves the lowest computational cost, suggesting higher efficiency. The other models, which gradually rise in cost from SAWL-Net to ResMTUNet and ES-CNN, are in the middle of these two extremes. In comparison to current methods, the proposed methodology shows improved scalability and decreased computing overhead.

**Fig. 20:**
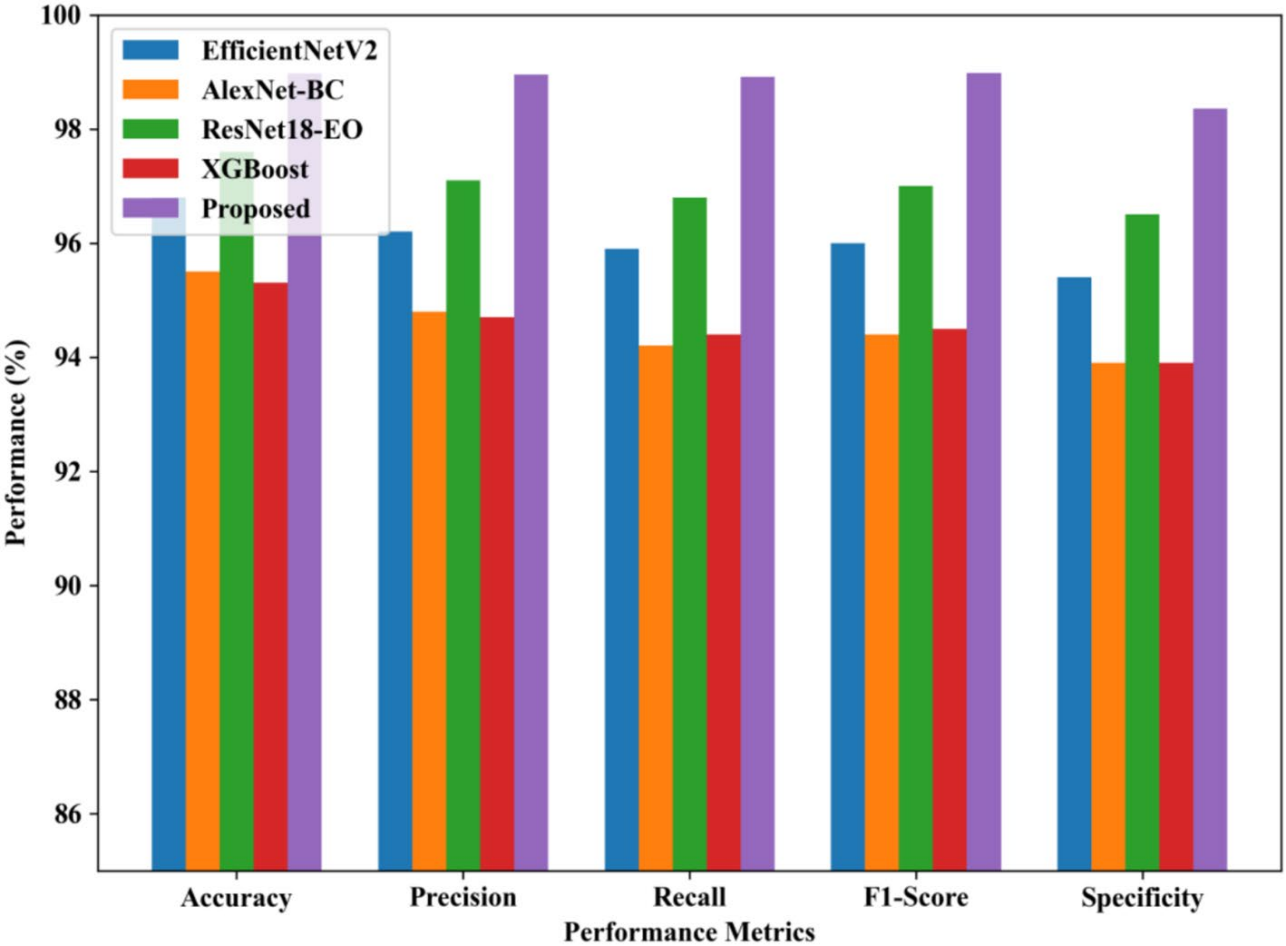
Performance Comparison of Proposed over Existing Methods.

The proposed approach clearly outperforms EfficientNetV2^32^, AlexNet-BC^33^, ResNet18-EO^34^, and XGBoost^35^ in all evaluation criteria, as shown in Fig. 20. With an accuracy of almost 99.2%, the proposed approach outperforms EfficientNetV2 at about 96.8%, ResNet18-EO at about 96.0%, AlexNet-BC at about 95.4%, and XGBoost at about 95.2%. The proposed approach achieves about 98.9% precision, while ResNet18-EO, EfficientNetV2, AlexNet-BC, and XGBoost record about 97.1%, 96.2%, and 94.8%, respectively. With a recall of almost 98.8%, the proposed strategy clearly outperforms ResNet18-EO, which has a recall of about 96.8%, and the other models, which have a recall of less than 96%.

**Fig. 21:**
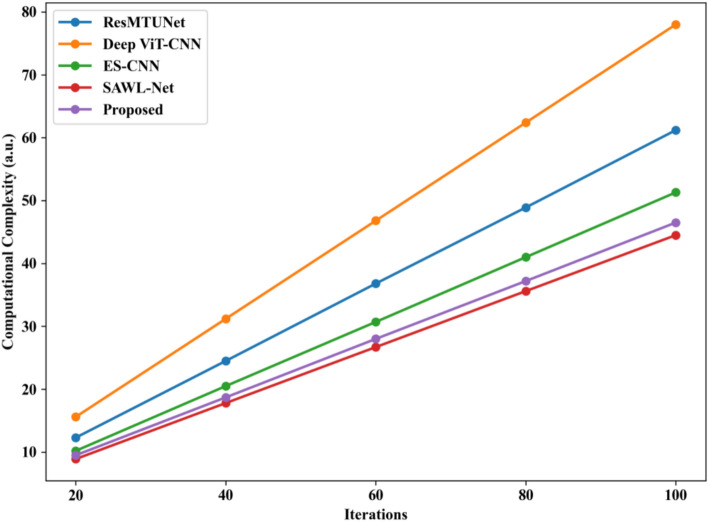
Computational Complexity analysis.

Figure 21 shows the computational complexity analysis. According to the analysis, the proposed BrCTransNet achieves high accuracy for breast cancer classification at a cheap computational cost. According to the computational complexity analysis, the suggested BrCTransNet maintains a low computational cost while classifying breast cancer with good accuracy. Compared to Deep ViT-CNN (78 a.u.) and ResMTUNet (61 a.u.), the proposed approach uses only about 46 a.u. at 100 iterations. This demonstrates that ChStF hyperparameter adjustment does not result in significant overhead. All things considered, the proposed approach offers a scalable and effective way to diagnose breast cancer with less computational strain.

**Fig. 22:**
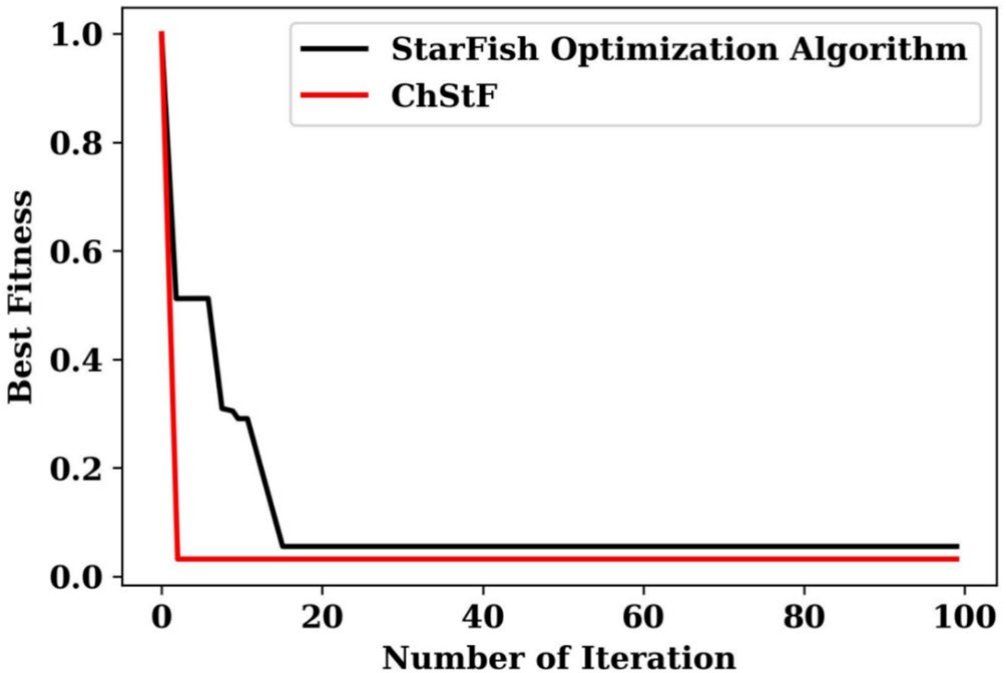
Analysis based on Convergence.

**Fig. 23:**
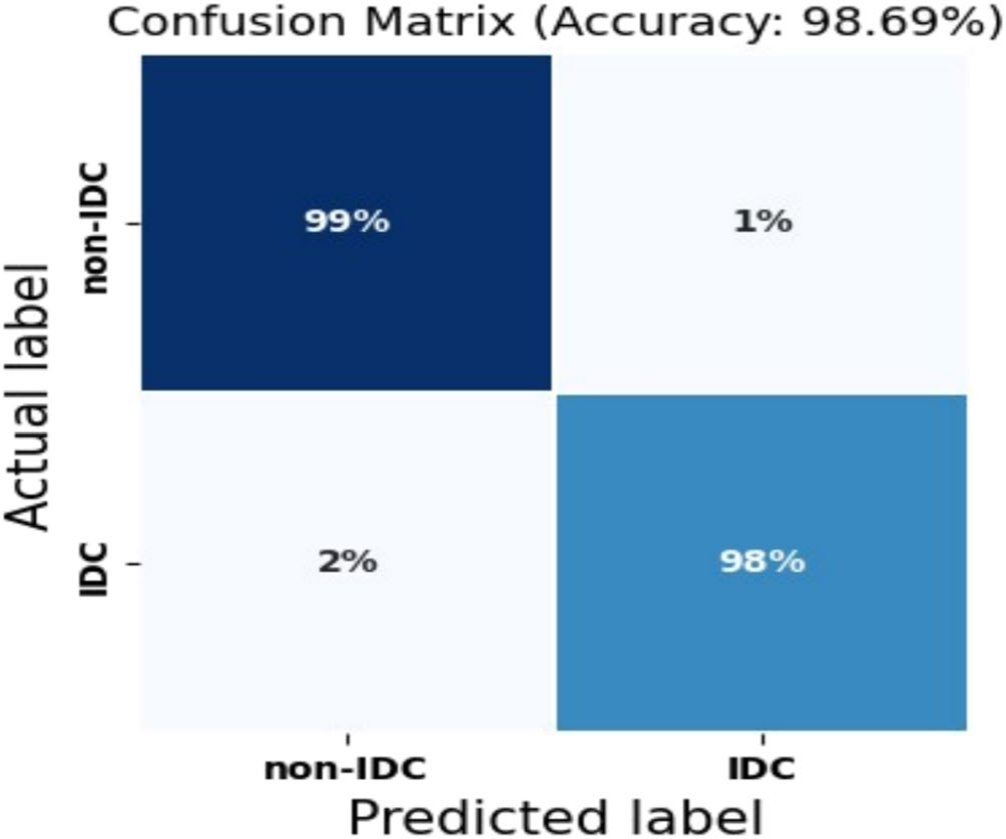
Analysis based on Confusion Matrix.

Figure 22 illustrates the convergence analysis, wherein ChStF algorithm introduces Chebyshev chaotic sequences to enhance diversification diversity and convergence speed and avoids premature convergence and local optima entrapment. It enables the model to converge faster compared to the traditional StarFish optimization algorithm. Figure 23 illustrates the confusion matrix that demonstrates the enhanced accuracy with minimal false classification of breast cancer.

**Comparative discussion**: Table 9 provides the comparative discussion based on the best outcome derived by the breast cancer classification models. The accuracy accomplished by the proposed method is 98.97%, which is 12.45%, 8.45%, 9.99% and 4.22% enhanced outcome compared to ResMTUNet, Deep ViT-CNN, ES-CNN, and SAWL-Net methods. The precision accomplished by the proposed method is 98.86%, which is 12.06%, 7.82%, 8.94%, and 3.57% enhanced outcome compared to ResMTUNet, Deep ViT-CNN, ES-CNN, and SAWL-Net methods. The recall accomplished by the proposed method is 98.91%, which is 11.45%, 8.36%, 11.25%, and 4.59% enhanced outcome compared to ResMTUNet, Deep ViT-CNN, ES-CNN, and SAWL-Net methods. The F1-Score accomplished by the proposed method is 98.98%, which is 15.06%, 10.86%, 12.86%, and 4.03% enhanced outcome compared to ResMTUNet, Deep ViT-CNN, ES-CNN, and SAWL-Net methods. The specificity accomplished by the proposed method is 98.36%, which is 11.85%, 5.84%, 6.97%, and 4.31% enhanced outcome compared to ResMTUNet, Deep ViT-CNN, ES-CNN, and SAWL-Net methods. The MSE accomplished by the proposed method is 0.0103, which is 92.32%, 89.08%, 90.61%, and 80.28% enhanced outcome compared to ResMTUNet, Deep ViT-CNN, ES-CNN, and SAWL-Net methods.

**Table 9:** Comparative discussion.

Metrics/ methods	ResMTUNet	Deep ViT-CNN	ES-CNN	SAWL-Net	Proposed
Accuracy	86.65	90.61	89.09	94.80	98.97
Precision	86.93	91.13	90.02	95.32	98.86
Recall	87.59	90.64	87.78	94.37	98.91
F1-score	84.08	88.23	86.25	95.00	98.98
Specificity	86.71	92.61	91.51	94.12	98.36
MSE	0.1335	0.0939	0.1091	0.0520	0.0103

The enhanced classification capability of proposed BrCTransNet model is achieved based on all assessment measures. Here, preprocessing techniques focus on both noise reduction and edge preservation, ensuring that subsequent analysis is based on clean, detail-rich image data. The refined input is then processed through deep hierarchical feature extraction facilitated by S2RMANet, which captures complex and relevant patterns within the images. Then, OPATransNet that allows the model to selectively focus on the most critical features for improving discriminative power. Finally, intelligent optimization using ChStF fine-tunes the model’s parameters that ensure optimal performance.

## Conclusion

This research presented the BrCTransNet framework, which integrates a thorough workflow of hierarchical feature extraction, noise-free preprocessing, and optimized transformer-based classification for reliable and accurate breast cancer classification. The Adaptive Gaussian Bilateral (AGB) filter is used to reduce noise and maintain important edges in the input histopathology images. The S2RMANet then extracts rich sequential spatial and multi-scale contextual characteristics, capturing long-range dependencies as well as local textures. After that, these features are categorized using OPATransNet, which improves discriminative global–local feature representation by adopting a polarized self-attention mechanism. By preventing local minima trapping, accelerating convergence, and optimizing the loss function, the ChStF algorithm significantly refines the model. Experimental evaluation on the Breast Histopathology Images dataset demonstrates that the proposed BrCTransNet model achieves an accuracy of 98.97%, precision of 98.86%, recall of 98.91%, F1-score of 98.98%, and specificity of 98.36%, with a minimal mean squared error (MSE) of 0.0103 compared to existing methods. High classification performance is ensured by the proposed hybrid architecture, which successfully balances local and global feature extraction ensures fast convergence and improves diagnostic reliability.

### Limitations and future work

The suggested BrCTransNet has several drawbacks despite its strong performance and high accuracy in classifying breast cancer. The model’s clinical dependability needs to be externally validated on several cohorts from various hospitals and scanners, as it was first assessed on a single public dataset (BreakHis/Kaggle). Second, the rising complexity of the model creates computational difficulties, especially when it comes to deployment in contexts with limited resources. Real-time application may be limited by the high memory and processing power requirements of training and inference. In order to overcome these constraints, future research will concentrate on optimizing the model’s computational footprint for edge-device compatibility, expanding its evaluation to multi-institutional datasets for reliable external validation, and investigating its use in multimodal diagnostic fusion and multi-class breast cancer subtyping. In order to lower computational requirements while maintaining diagnostic accuracy, methods like model pruning, quantization, or knowledge distillation will also be examined.

## Data Availability

The datasets used and/or analysed during the current study are available from the corresponding author on reasonable request.

## Declarations

### Dual publication

No.

### Third party material

No.
